# Binding of Gtf2i-β/δ transcription factors to the *ARMS2* gene leads to increased circulating HTRA1 in AMD patients and *in vitro*

**DOI:** 10.1016/j.jbc.2021.100456

**Published:** 2021-02-24

**Authors:** Yang Pan, Daisuke Iejima, Mao Nakayama, Akiko Suga, Toru Noda, Inderjeet Kaur, Taraprasad Das, Subhabrata Chakrabarti, Robyn H. Guymer, Margaret M. DeAngelis, Megumi Yamamoto, Paul N. Baird, Takeshi Iwata

**Affiliations:** 1 Division of Molecular and Cellular Biology, National Institute of Sensory Organs, National Hospital Organization Tokyo Medical Center, Tokyo, Japan; 2 Division of Ophthalmology, National Hospital Organization Tokyo Medical Center, Tokyo, Japan; 3 Kallam Anji Reddy Molecular Genetics lab, Prof Brien Holden Eye Research Centre, L. V. Prasad Eye Institute, Hyderabad, India; 4 Centre for Eye Research Australia, University of Melbourne, Royal Victorian Eye & Ear Hospital, East Melbourne, Victoria, Australia; 5 Department of Surgery, Ophthalmology, Faculty of Medicine, Dentistry and Health Sciences, The University of Melbourne, East Melbourne, Victoria, Australia; 6 Department of Ophthalmology and Ira G. Ross Eye Institute, Jacobs School of Medicine and Biomedical Sciences, University at Buffalo, The State University of New York, Buffalo, New York, USA; 7 JAC Ltd, Tokyo, Japan

**Keywords:** age-related macular degeneration, HTRA1, ARMS2, promoter, secretion, ALK5, activin receptor-like kinase 5, AMD, age-related macular degeneration, ARMS2, *Age-Related Maculopathy Susceptibility 2*, CFH, complement factor H, CNV, choroidal neovascularization, EF1, elongation factor 1, FCS, fetal calf serum, GA, geographic atrophy, Gtf2i, general transcription factor Iii, GWAS, genome-wide association studies, H&E, hematoxylin and eosin, hnRNP-K, heterogeneous nuclear ribonucleoprotein K, HTRA1, *High Temperature Requirement A1*, in/del, insertion/deletion, iPSCs, induced pluripotent stem cells, LD, linkage disequilibrium, OCT, optical coherence tomography, PCV, polypoidal choroidal vasculopathy, RT-PCR, reverse transcription-PCR, TGF-β2, transforming growth factor-β2, UTR, untranslated regions, VEGF, vascular endothelial growth factor, WB, western blotting

## Abstract

The disease-initiating molecular events for age-related macular degeneration (AMD), a multifactorial retinal disease affecting many millions of elderly individuals worldwide, are still unknown. Of the over 30 risk and protective loci so far associated with AMD through whole genome-wide association studies (GWAS), the *Age-Related Maculopathy Susceptibility 2* (*ARMS2*) gene locus represents one of the most highly associated risk regions for AMD. A unique insertion/deletion (in/del) sequence located immediately upstream of the *High Temperature Requirement A1* (*HTRA1*) gene in this region confers high risk for AMD. Using electrophoretic mobility shift assay (EMSA), we identified that two Gtf2i-β/δ transcription factor isoforms bind to the cis-element 5′- ATTAATAACC-3′ contained in this in/del sequence. The binding of these transcription factors leads to enhanced upregulation of transcription of the secretory serine protease HTRA1 in transfected cells and AMD patient-derived induced pluripotent stem cells (iPSCs). Overexpression of Htra1 in mice using a CAG-promoter demonstrated increased blood concentration of Htra1 protein, caused upregulation of vascular endothelial growth factor (VEGF), and produced a choroidal neovascularization (CNV)-like phenotype. Finally, a comparison of 478 AMD patients to 481 healthy, age-matched controls from Japan, India, Australia, and the USA showed a statistically increased level of secreted HTRA1 blood concentration in AMD patients compared with age-matched controls. Taken together, these results suggest a common mechanism across ethnicities whereby increased systemic blood circulation of secreted serine protease HTRA1 leads to subsequent degradation of Bruch's membrane and eventual CNV in AMD.

AMD is a multifactorial eye disease that affects approximately 8.7% of the world's population. The number of individuals with this disease is expected to increase from 196 million in 2020 to 288 million in 2040 (1). The two main end-stage disease phenotypes of AMD are classified as dry AMD (geographic atrophy, GA) and wet AMD (choroidal neovascularization, CNV) (2). The incidence and distribution of dry and wet AMD differ between ethnic groups (3). GA occurs more often in the Caucasian population, and CNV occurs more often in the East Asian population (4). Genetic, age, and environmental risk factors affect AMD (3, 5, 6, 7). The International AMD Genomics Consortium has reported more than 34 distinct loci resulting in 52 independently associated common and rare AMD variants through GWAS (6, 8). These include significant association with many complement genes, including *CFH* (8), *CFI* (9), *CFB* (10), and *C3* (11). However, the most highly associated locus is the AMD-associated linkage disequilibrium (LD) block, which stretches approximately 10.5 Kbp between the *ARMS2* and *HTRA1* genes on chromosome 10. Within this unique LD-block is a 54 bp nucleotide insertion/443 bp deletion “in/del” (GenBank: EU427539) located in the 3-prime untranslated regions (3′UTR) of the *ARMS2* gene and significantly associated with both GA and CNV (7, 12, 13). The heterozygous in/del allele frequency has been shown to be higher in AMD patients (1.49–2.24-fold) compared with controls in various populations from Germany, the USA, Northern Europe, Italy, China, Japan, Australia, and South India (13). The homozygous in/del allele frequency is much higher in AMD patients compared with controls in Australia and India by 51.38-fold and 2.77-fold, respectively (13). Insertion of the 54 bp in/del sequence has been shown to lead to rapid mRNA turnover with homozygote carriers showing no expression of ARMS2 (14). We also previously described that only marginal promoter activity was observed for ARMS2 (15).

We and others have previously demonstrated that the in/del sequence along with a downstream promoter region can significantly induce *HTRA1* transcription in transfected 661W (15) and Y79 (16) cell lines. Detailed analysis of this region by luciferase assay has shown that the in/del portion of the sequence is especially critical to induce promoter activity, and electrophoretic mobility shift assay (EMSA) detected an unknown transcriptional activator(s) binding to this in/del sequence (15). Induction of HTRA1 transcription was identified in iPSCs derived from CNV AMD patients with the in/del sequence (15). Our transgenic mouse overexpressing Htra1 through a ubiquitous CAG promoter (CAG-*Htra1* Tg mouse) showed a CNV-like phenotype after 12 months (17) while others have, using RPE-specific promoters, demonstrated a polypoidal choroidal vasculopathy (PCV)-like phenotype but not CNV (18, 19).

HTRA1 is a secreted serine protease known to inhibit transforming growth factor-β2 (TGF-β2) signaling, an important regulator of angiogenesis by degradation (5). The aqueous levels of active TGF-β2 are lower in the retina of AMD patients compared with controls (20), and TGF-β2 knockout mice exhibit abnormal ocular morphogenesis phenotypes in the retina (21). Considering the crucial roles of TGF-β2/activin receptor-like kinase 5 (ALK5)/SMAD2/3 signaling in neovascularization, unraveling the molecular mechanisms of HTRA1 upregulation involving the in/del sequence is important in understanding the development of CNV in AMD.

In this study, we identified the transcription factors binding to the in/del of the 3′UTR region of *ARMS2*. We also observed that overexpression of these transcription factors induced *HTRA1* transcription in iPSCs derived from AMD patients as well as in the blood of CNV mice ubiquitously overexpressing *Htra1*. Further study demonstrated a significant increase of HTRA1 protein concentration in blood in both GA and CNV AMD patients carrying the in/del sequence compared with age-matched controls, indicating that AMD is also associated with an increase of blood circulating HTRA1.

## Results

### Identification of the in/del binding transcription factors responsible for induction of *HTRA1* transcription

**Figure 1:**
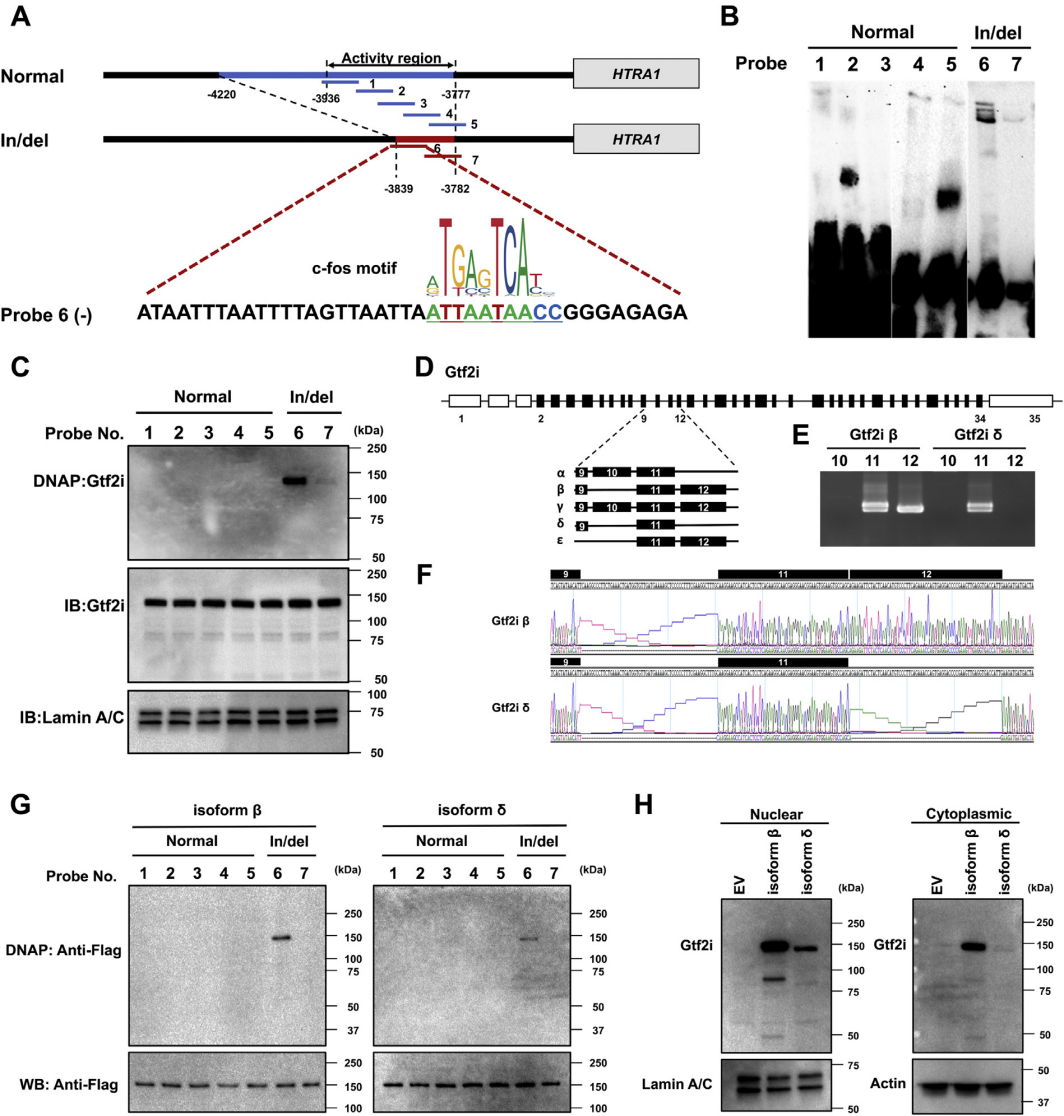
**In/del binding transcription factor protein**. *A*, schematic illustration of the double-stranded DNA probes for EMSA (gel electrophoresis mobility shift assay) in the region upstream of the HTRA1 activity coding region. Linking c-fos transcription factor to in/del-6. Profile of the c-fos transcription factor-binding sequence (ID: MA0099.3 from the JASPAR database). *B*, EMSA was performed to determine the in/del binding activity protein. In total, 50 μl nuclear protein from 661W cells was incubated with 100 pmol biotin-labeled Double-stranded DNA probes and analyzed on a 7.5% EMSA gel. Bands of interest were cut out and processed for LC-MS/MS analysis. *C*, Gtf2i-DNA probes binding test. The binding ability between Gtf2i and the in/del region was confirmed by WB. In/del DNA probes 6 and 7 were detected by an anti-Gtf2i antibody. Detection of Lamin A/C was used as an internal control. *D*, gene structure of Murine Gtf2i. Coding exons are depicted as *black boxes* and noncoding exons are in *blank boxes*. Five isoforms signify various alternatively spliced isoforms with exon 9, 10, 11, and 12 indicated. In 661W cells, there are only isoforms β and δ present by TA cloning (*E*, *F*). *G*, Gtf2i β/δ -DNA probes binding test. Both Gtf2i β and δ bind to the in/del-6 probe by WB. *H*, expression of Gtf2i isoform β and δ in nuclear or cytoplasmic extracts. Vector of Gtf2i isoform β or δ was transfected into 661W cells, followed by WB. Gtf2i isoform β was expressed in both nuclear and cytoplasmic extracts. However, isoform δ was only detected in nuclear but cytoplasmic extracts.

Isolation of in/del binding transcription factor(s) was performed by EMSA using the nuclear extract from 661W cell line (Fig. 1, *A* and *B*). Of all the listed in/del interacting proteins, multifunctional general transcription factor IIi (Gtf2i) was identified with the most number of peptide hits (Table 1). This transcription factor commonly targets the *c-fos cis*-element binding nucleotide sequence (22, 23, 24, 25). A consensus *c-fos cis-*element 5′-ATTAATAACC-3′ was identified in the in/del sequence by JASPAR 2018 (Figs. 1*A* and S1). Gtf2i was the only transcription factor among all listed peptides, where this *cis-element* binding sequence was found within the in/del sequence. An anti-Gtf2i antibody was used to confirm Gtf2i binding to DNA probes 6 and 7 on the EMSA blot during western blotting (WB) that confirmed the single Gtf2i signal (Fig. 1*C*). The elongation factor 1 (EF1) and heterogeneous nuclear ribonucleoprotein K (hnRNP-K) isolated by non-in/del sequence DNA probes 1–5 were not detected by WB (Fig. S2). Each isoform of Gtf2i (α, β, γ, δ, ε) (26) was TA cloned and assayed for binding with in/del probes by WB (Fig. 1, *D–F*). Only two isoforms (Gtf2i-β and -δ) were confirmed binding to DNA probe 6 (Fig. 1*G*). Isoform Gtf2i-β is expressed in both the nucleus and cytoplasm in 661W cells, while isoform Gtf2i-δ is detected exclusively in the nucleus of the 661W cell line (Fig. 1*H*).

**Table 1:** List of in/del and normal sequence binding proteins

In/del sequence specific binding proteins										
No.	Identified proteins (24/275)	Accession number	Molecular weight (kDa)	Taxonomy	In/del 001	In/del 002	In/del 003	Normal 001	Normal 002	Normal 003
1	General transcription factor II-I OS=*Mus musculus* GN=Gtf2i PE=1 SV=3	GTF2I_MOUSE	112	*Mus musculus*	7	6	4	0	0	0
2	Splicing factor 3A subunit 1 OS=*Mus musculus* GN=Sf3a1 PE=1 SV=1	SF3A1_MOUSE	89	*Mus musculus*	4	3	4	0	0	0
3	U2 snRNP-associated SURP motif-containing protein OS=*Mus musculus* GN=U2surp PE=2 SV=3	SR140_MOUSE	118	*Mus musculus*	4	3	1	0	0	0
4	Cleavage stimulation factor subunit 1 OS=*Mus musculus* GN=Cstf1 PE=2 SV=1	CSTF1_MOUSE	48	*Mus musculus*	4	2	4	0	0	0
5	Pre-mRNA-processing factor 19 OS=*Mus musculus* GN=Prpf19 PE=1 SV=1	PRP19_MOUSE	55	*Mus musculus*	3	5	7	0	0	0
6	U1 small nuclear ribonucleoprotein A OS=*Mus musculus* GN=Snrpa PE=2 SV=3	SNRPA_MOUSE	32	*Mus musculus*	3	3	2	0	0	0
7	THO complex subunit 4 OS=*Mus musculus* GN=Alyref PE=1 SV=3	THOC4_MOUSE	27	*Mus musculus*	3	2	3	0	0	0
8	ATP-dependent RNA helicase DDX42 OS=*Mus musculus* GN=Ddx42 PE=1 SV=3	DDX42_MOUSE	102	*Mus musculus*	3	2	3	0	0	0
9	78 kDa glucose-regulated protein OS=*Mus musculus* GN=Hspa5 PE=1 SV=3	GRP78_MOUSE	72	*Mus musculus*	3	1	1	0	0	0
10	Splicing factor 45 OS=*Mus musculus* GN=Rbm17 PE=1 SV=1	SPF45_MOUSE	45	*Mus musculus*	2	3	3	0	0	0
11	Flap endonuclease 1 OS=*Mus musculus* GN=Fen1 PE=1 SV=1	FEN1_MOUSE	42	*Mus musculus*	2	2	2	0	0	0
12	E3 ubiquitin/ISG15 ligase TRIM25 OS=*Mus musculus* GN=Trim25 PE=1 SV=2	TRI25_MOUSE	72	*Mus musculus*	2	1	2	0	0	0
13	PC4 and SFRS1-interacting protein OS=*Mus musculus* GN=Psip1 PE=1 SV=1	PSIP1_MOUSE	60	*Mus musculus*	1	3	2	0	0	0
14	Heterogeneous nuclear ribonucleoprotein D-like OS=*Mus musculus* GN=Hnrnpdl PE=1 SV=1	HNRDL_MOUSE	34	*Mus musculus*	1	2	2	0	0	0
15	Small nuclear ribonucleoprotein Sm D2 OS=*Mus musculus* GN=Snrpd2 PE=2 SV=1	SMD2_MOUSE	14	*Mus musculus*	1	2	1	0	0	0
16	H/ACA ribonucleoprotein complex subunit 1 OS=*Mus musculus* GN=Gar1 PE=2 SV=1	GAR1_MOUSE	23	*Mus musculus*	1	1	2	0	0	0
17	60S ribosomal protein L14 OS=*Mus musculus* GN=Rpl14 PE=1 SV=3	RL14_MOUSE	24	*Mus musculus*	1	1	2	0	0	0
18	40S ribosomal protein S25 OS=*Mus musculus* GN=Rps25 PE=1 SV=1	RS25_MOUSE	14	*Mus musculus*	1	1	2	0	0	0
19	ELAV-like protein 1 OS=*Mus musculus* GN=Elavl1 PE=1 SV=2	ELAV1_MOUSE	36	*Mus musculus*	1	1	2	0	0	0
20	Zinc finger CCCH-type antiviral protein 1 OS=*Mus musculus* GN=Zc3hav1 PE=1 SV=1	ZCCHV_MOUSE	107	*Mus musculus*	1	1	1	0	0	0
21	Structural maintenance of chromosomes flexible hinge domain-containing protein 1 OS=*Mus musculus* GN=Smchd1 PE=1 SV=2	SMHD1_MOUSE	226	*Mus musculus*	1	1	1	0	0	0
22	Cleavage stimulation factor subunit 2 OS=*Mus musculus* GN=Cstf2 PE=1 SV=2	CSTF2_MOUSE	61	*Mus musculus*	1	1	1	0	0	0
23	Protein SCAF8 OS=*Mus musculus* GN=Scaf8 PE=1 SV=1	SCAF8_MOUSE	140	*Mus musculus*	1	1	1	0	0	0
24	Phenylalanine--tRNA ligase alpha subunit OS=*Mus musculus* GN=Farsa PE=2 SV=1	SYFA_MOUSE	58	*Mus musculus*	1	1	1	0	0	0

Normal sequence-specific binding proteins										
No.	Identified Proteins (17/275)	Accession Number	Molecular Weight (KDa)	Taxonomy	In/del 001	In/del 002	In/del 003	Normal 001	Normal 002	Normal 003
1	Elongation factor 1-gamma OS=*Mus musculus* GN=Eef1g PE=1 SV=3	EF1G_MOUSE	50	*Mus musculus*	0	0	0	20	18	17
2	Heterogeneous nuclear ribonucleoprotein K OS=*Mus musculus* GN=Hnrnpk PE=1 SV=1	HNRPK_MOUSE	51	*Mus musculus*	0	0	0	16	14	15
3	Valine–tRNA ligase OS=*Mus musculus* GN=Vars PE=2 SV=1	SYVC_MOUSE	140	*Mus musculus*	0	0	0	24	15	21
4	Elongation factor 1-delta OS=*Mus musculus* GN=Eef1d PE=1 SV=3	EF1D_MOUSE	31	*Mus musculus*	0	0	0	16	13	13
5	Hemoglobin subunit beta-1 OS=*Mus musculus* GN=Hbb-b1 PE=1 SV=2	HBB1_MOUSE	16	*Mus musculus*	0	0	0	8	7	10
6	Elongation factor 1-beta OS=*Mus musculus* GN=Eef1b PE=1 SV=5	EF1B_MOUSE	25	*Mus musculus*	0	0	0	6	6	5
7	Kinectin OS=*Mus musculus* GN=Ktn1 PE=2 SV=1	KTN1_MOUSE	153	*Mus musculus*	0	0	0	3	6	2
8	Poly(rC)-binding protein 2 OS=*Mus musculus* GN=Pcbp2 PE=1 SV=1	PCBP2_MOUSE	38	*Mus musculus*	0	0	0	7	5	7
9	Hemoglobin subunit alpha OS=*Mus musculus* GN=Hba PE=1 SV=2	HBA_MOUSE	15	*Mus musculus*	0	0	0	4	4	4
10	Heterogeneous nuclear ribonucleoprotein L-like OS=*Mus musculus* GN=Hnrnpll PE=1 SV=3	HNRLL_MOUSE	64	*Mus musculus*	0	0	0	2	4	5
11	Myelin expression factor 2 OS=*Mus musculus* GN=Myef2 PE=1 SV=1	MYEF2_MOUSE	63	*Mus musculus*	0	0	0	4	1	1
12	Protein argonaute-2 OS=*Mus musculus* GN=Ago2 PE=1 SV=3	AGO2_MOUSE	97	*Mus musculus*	0	0	0	2	1	2
13	Keratin, type II cytoskeletal 2 epidermal OS=*Mus musculus* GN=Krt2 PE=1 SV=1	K22 E_MOUSE	71	*Mus musculus*	0	0	0	2	1	1
14	YTH domain-containing family protein 2 OS=*Mus musculus* GN=Ythdf2 PE=2 SV=1	YTHD2_MOUSE	62	unknown	0	0	0	1	1	2
15	Poly(rC)-binding protein 4 OS=*Mus musculus* GN=Pcbp4 PE=2 SV=1	PCBP4_MOUSE	41	*Mus musculus*	0	0	0	1	1	1
16	Transcriptional activator protein Pur-beta OS=*Mus musculus* GN=Purb PE=1 SV=3	PURB_MOUSE	34	*Mus musculus*	0	0	0	1	1	1
17	Core-binding factor subunit beta OS=*Mus musculus* GN=Cbfb PE=1 SV=1	PEBB_MOUSE	22	*Mus musculus*	0	0	0	1	1	1

Normal and in/del sequence common binding proteins										
No.	Identified Proteins (275)	Accession Number	Molecular Weight (kDa)	Taxonomy	In/del 001	In/del 002	In/del 003	Normal 001	Normal 002	Normal 003
1	Heterogeneous nuclear ribonucleoprotein A1 OS=*Mus musculus* GN=Hnrnpa1 PE=1 SV=2	ROA1_MOUSE	34	*Mus musculus*	19	16	16	17	14	17
2	Elongation factor 1-alpha 1 OS=*Mus musculus* GN=Eef1a1 PE=1 SV=3	EF1A1_MOUSE	50	*Mus musculus*	11	5	7	15	14	14
3	Heterogeneous nuclear ribonucleoproteins A2/B1 OS=*Mus musculus* GN=Hnrnpa2b1 PE=1 SV=2	ROA2_MOUSE	37	*Mus musculus*	12	12	12	10	11	10
4	Nucleolin OS=*Mus musculus* GN=Ncl PE=1 SV=2	NUCL_MOUSE	77	*Mus musculus*	18	20	19	6	5	3
5	Cytoskeleton-associated protein 5 OS=*Mus musculus* GN=Ckap5 PE=2 SV=1	CKAP5_MOUSE	226	*Mus musculus*	15	13	16	8	6	13
6	Non-POU domain-containing octamer-binding protein OS=*Mus musculus* GN=Nono PE=1 SV=3	NONO_MOUSE	55	*Mus musculus*	10	12	14	8	8	9
7	RNA-binding protein FUS OS=*Mus musculus* GN=Fus PE=2 SV=1	FUS_MOUSE	53	*Mus musculus*	8	7	8	6	8	7
8	Heterogeneous nuclear ribonucleoprotein A3 OS=*Mus musculus* GN=Hnrnpa3 PE=1 SV=1	ROA3_MOUSE	40	*Mus musculus*	10	10	10	7	8	8
9	Septin-11 OS=*Mus musculus* GN=Sept11 PE=1 SV=4	SEP11_MOUSE	50	*Mus musculus*	10	11	12	10	10	8
10	Pyruvate kinase PKM OS=*Mus musculus* GN=Pkm PE=1 SV=4	KPYM_MOUSE	58	*Mus musculus*	8	6	6	17	19	16
11	Splicing factor, proline- and glutamine-rich OS=*Mus musculus* GN=Sfpq PE=1 SV=1	SFPQ_MOUSE	75	*Mus musculus*	10	12	12	6	5	5
12	Heterogeneous nuclear ribonucleoprotein A/B OS=*Mus musculus* GN=Hnrnpab PE=1 SV=1	ROAA_MOUSE	31	*Mus musculus*	7	9	8	9	8	8
13	Septin-7 OS=*Mus musculus* GN=Sept7 PE=1 SV=1	SEPT7_MOUSE	51	*Mus musculus*	11	13	11	10	9	11
14	Polypyrimidine tract-binding protein 1 OS=*Mus musculus* GN=Ptbp1 PE=1 SV=2	PTBP1_MOUSE	56	*Mus musculus*	3	4	5	10	9	11
15	Probable ATP-dependent RNA helicase DDX5 OS=*Mus musculus* GN=Ddx5 PE=1 SV=2	DDX5_MOUSE	69	*Mus musculus*	12	11	13	4	4	6
16	Heterogeneous nuclear ribonucleoprotein A0 OS=*Mus musculus* GN=Hnrnpa0 PE=1 SV=1	ROA0_MOUSE	31	*Mus musculus*	7	9	8	8	7	7
17	Septin-2 OS=*Mus musculus* GN=Sept2 PE=1 SV=2	SEPT2_MOUSE	42	*Mus musculus*	8	10	10	5	7	6
18	Zinc finger X-linked protein ZXDB OS=*Mus musculus* GN=Zxdb PE=2 SV=1	ZXDB_MOUSE	90	unknown	5	4	3	2	1	2
19	YTH domain-containing family protein 3 OS=*Mus musculus* GN=Ythdf3 PE=1 SV=2	YTHD3_MOUSE	64	*Mus musculus*	8	7	7	8	9	9
20	ATP-dependent RNA helicase DDX1 OS=*Mus musculus* GN=Ddx1 PE=1 SV=1	DDX1_MOUSE	83	*Mus musculus*	3	4	5	11	7	10
21	Heterogeneous nuclear ribonucleoprotein Q OS=*Mus musculus* GN=Syncrip PE=1 SV=2	HNRPQ_MOUSE	70	*Mus musculus*	10	10	11	1	1	2
22	RNA-binding protein EWS OS=*Mus musculus* GN=Ewsr1 PE=1 SV=2	EWS_MOUSE	68	*Mus musculus*	5	4	4	6	5	4
23	Septin-9 OS=*Mus musculus* GN=Sept9 PE=1 SV=1	SEPT9_MOUSE	66	*Mus musculus*	5	5	6	6	6	6
24	KH domain-containing, RNA-binding, signal transduction-associated protein 1 OS=*Mus musculus* GN=Khdrbs1 PE=1 SV=2	KHDR1_MOUSE	48	*Mus musculus*	7	6	8	2	2	2
25	Splicing factor 3B subunit 3 OS=*Mus musculus* GN=Sf3b3 PE=2 SV=1	SF3B3_MOUSE	136	*Mus musculus*	11	11	15	2	1	1
26	Heterogeneous nuclear ribonucleoprotein D0 OS=*Mus musculus* GN=Hnrnpd PE=1 SV=2	HNRPD_MOUSE	38	*Mus musculus*	4	7	6	5	5	6
27	Heterogeneous nuclear ribonucleoprotein M OS=*Mus musculus* GN=Hnrnpm PE=1 SV=3	HNRPM_MOUSE	78	*Mus musculus*	6	4	4	6	7	7
28	Keratin, type I cytoskeletal 10 OS=*Mus musculus* GN=Krt10 PE=1 SV=3	K1C10_MOUSE	58	*Mus musculus*	5	2	3	6	6	8
29	Poly(rC)-binding protein 1 OS=*Mus musculus* GN=Pcbp1 PE=1 SV=1	PCBP1_MOUSE	37	*Mus musculus*	4	2	3	8	10	12
30	ADP/ATP translocase 2 OS=*Mus musculus* GN=Slc25a5 PE=1 SV=3	ADT2_MOUSE	33	*Mus musculus*	7	7	8	2	5	5
31	tRNA-splicing ligase RtcB homolog OS=*Mus musculus* GN=Rtcb PE=2 SV=1	RTCB_MOUSE	55	*Mus musculus*	5	2	1	9	6	3
32	Leucine-rich repeat-containing protein 59 OS=*Mus musculus* GN=Lrrc59 PE=1 SV=1	LRC59_MOUSE	35	*Mus musculus*	9	9	9	2	1	2
33	UPF0568 protein C14orf166 homolog OS=*Mus musculus* PE=2 SV=1	CN166_MOUSE	28	*Mus musculus*	3	4	4	6	6	5
34	Probable 28S rRNA (cytosine-C(5))-methyltransferase OS=*Mus musculus* GN=Nop2 PE=1 SV=1	NOP2_MOUSE	87	*Mus musculus*	7	7	6	5	5	6
35	Splicing factor 3B subunit 1 OS=*Mus musculus* GN=Sf3b1 PE=1 SV=1	SF3B1_MOUSE	146	*Mus musculus*	7	3	5	1	2	2
36	Anionic trypsin-2 OS=*Mus musculus* GN=Prss2 PE=2 SV=1	TRY2_MOUSE	26	*Mus musculus*	1	1	1	1	1	1
37	Keratin, type II cytoskeletal 79 OS=*Mus musculus* GN=Krt79 PE=2 SV=2	K2C79_MOUSE	58	*Mus musculus*	1	1	1	2	3	2
38	Serum albumin OS=*Mus musculus* GN=Alb PE=1 SV=3	ALBU_MOUSE	69	*Mus musculus*	2	3	2	3	3	4
39	Ras GTPase-activating protein-binding protein 2 OS=*Mus musculus* GN=G3bp2 PE=1 SV=2	G3BP2_MOUSE	54	*Mus musculus*	2	2	3	2	4	3
40	Caprin-1 OS=*Mus musculus* GN=Caprin1 PE=1 SV=2	CAPR1_MOUSE	78	*Mus musculus*	4	5	2	2	3	3
41	Histone H1.1 OS=*Mus musculus* GN=Hist1h1a PE=1 SV=2	H11_MOUSE	22	*Mus musculus*	2	2	2	3	2	3
42	Actin, cytoplasmic 1 OS=*Mus musculus* GN=Actb PE=1 SV=1	ACTB_MOUSE	42	*Mus musculus*	1	2	2	4	3	3
43	Keratin, type II cytoskeletal 5 OS=*Mus musculus* GN=Krt5 PE=1 SV=1	K2C5_MOUSE	62	*Mus musculus*	2	3	2	2	2	2
44	60S ribosomal protein L30 OS=*Mus musculus* GN=Rpl30 PE=2 SV=2	RL30_MOUSE	13	*Mus musculus*	3	3	5	3	1	2
45	DAZ-associated protein 1 OS=*Mus musculus* GN=Dazap1 PE=2 SV=2	DAZP1_MOUSE	43	*Mus musculus*	3	3	2	1	1	1
46	RNA-binding protein 39 OS=*Mus musculus* GN=Rbm39 PE=1 SV=2	RBM39_MOUSE	59	*Mus musculus*	3	3	3	2	4	4
47	Histone H1.2 OS=*Mus musculus* GN=Hist1h1c PE=1 SV=2	H12_MOUSE	21	*Mus musculus*	2	2	2	2	2	3
48	YTH domain-containing family protein 1 OS=*Mus musculus* GN=Ythdf1 PE=2 SV=1	YTHD1_MOUSE	61	*Mus musculus*	3	1	1	3	3	2
49	Putative RNA-binding protein 3 OS=*Mus musculus* GN=Rbm3 PE=2 SV=1	RBM3_MOUSE	17	*Mus musculus*	2	3	2	2	3	1
50	Hyaluronan mediated motility receptor OS=*Mus musculus* GN=Hmmr PE=1 SV=4	HMMR_MOUSE	92	*Mus musculus*	2	1	2	3	1	1
51	Keratin, type II cytoskeletal 8 OS=*Mus musculus* GN=Krt8 PE=1 SV=4	K2C8_MOUSE	55	*Mus musculus*	1	1	1	1	1	1
52	60S ribosome subunit biogenesis protein NIP7 homolog OS=*Mus musculus* GN=Nip7 PE=2 SV=1	NIP7_MOUSE	20	*Mus musculus*	1	3	2	1	1	2

Protein threshold:99.9%; peptide threshold: 95%.

The proteins of interest showing biological significance are underlined.

**Figure 2:**
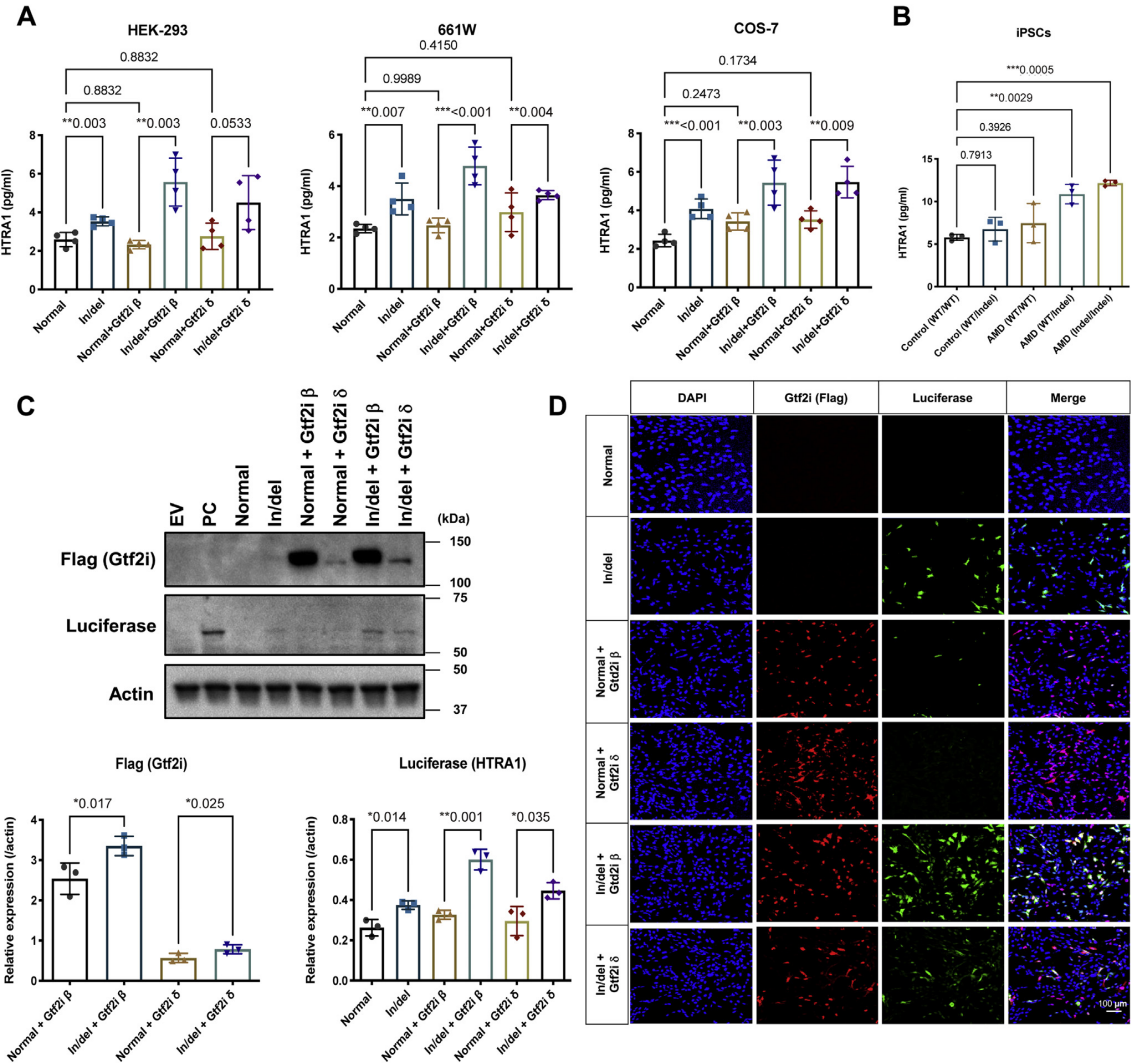
**Function analysis for Gtf2i and HTRA1.***A*, HTRA1 in/del and normal with or without Gtf2i β/δ vectors were transfected in HEK-293, 661W and COS-7 cells, respectively, following ELISA. Presence of the in/del significantly enhanced HTRA1 secretion and Gtf2i β/δ promoted enhanced efficiency with indel-HTRA1 but not the normal-HTRA1 in all cell lines. *B*, secretion of HTRA1 in human iPSCs derived from AMD patients. iPSCs were derived from individuals with normal *versus* in/del transcription regulators, followed by ELISA. Transfection of 661W cells with the Gtf2i β or δ expression vectors significantly enhanced in/del-Luciferase transcription comparing with normal. Representative WB (*C*) and immunocytochemistry (*D*) results are shown. Throughout, the results are expressed as the mean ± SEM. The *p* value was obtained by Student's *t* test.

Finally, HTRA1 expression and secretion were measured by cotransfection of either the in/del-promoter-HTRA1/luciferase cDNA or normal-promoter-HTRA1/luciferase cDNA constructs with either Gtf2i-β cDNA or Gtf2i-δ cDNA or both constructs transfected into the 661W cell line. Overexpression of Gtf2i-β and Gtf2i-δ independently enhanced HTRA1 transcription and protein secretion by the presence of the in/del-promoter-HTRA1/luciferase cDNA construct compared with the normal-promoter- HTRA1/luciferase cDNA construct (Fig. 2, *A*, *C*, and *D* and Fig. S3). These results suggested that *HTRA1* promoter activity is upregulated by the binding of two transcription factors Gtf2i β and Gtf2i δ to indel, providing new insight for the molecular mechanism of HTRA1 transcription by highly AMD-associated in/del haplotype.

### Induced HTRA1 protein secretion from in/del-promoter-HTRA1 cDNA transfected cells and from AMD patient iPSCs with the in/del

The expression vector constructed with HTRA1 cDNA along with its promoter and upper regulatory region with and without the in/del haplotype sequence (in/del-promoter-HTRA1) was transfected into 661W, COS-7, and HEK-293 cell lines resulting in an elevation of secreted HTRA1 protein in the cell culture medium by ELISA (Fig. 2*A*). Moreover, elevation of secreted HTRA1 protein was observed in the cell culture medium of iPS cells derived from AMD patients with the in/del haplotype compared with iPS cells from general controls (Fig. 2*B*).

### Induced blood Htra1 protein concentration in *HtrA1* Tg mice with CNV

**Figure 3:**
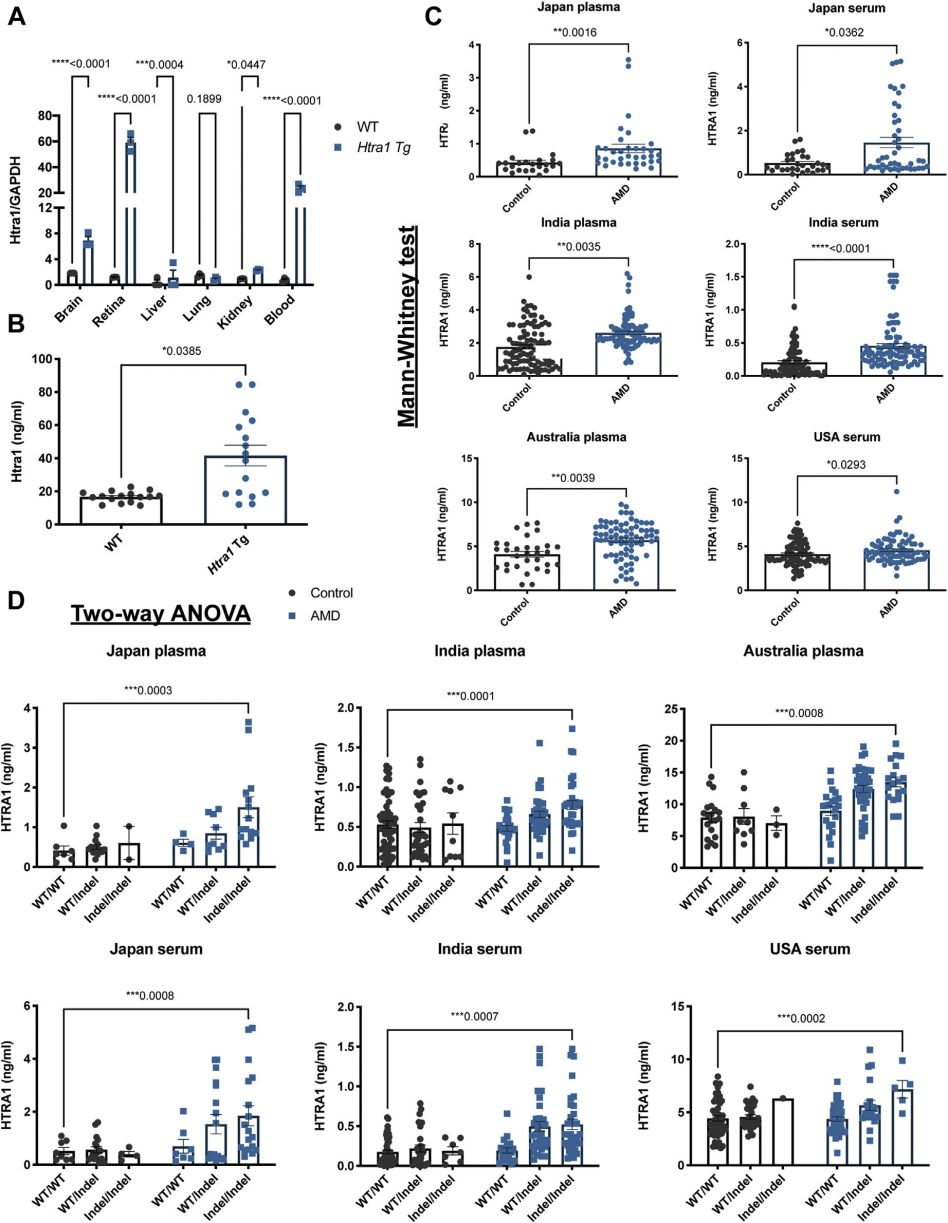
**Comparison of Htra1 expression and secretion in mouse and human.***A*, quantitative real-time PCR determination of *Htra1* mRNA levels from total RNA isolated from the brains, retina, liver, lung, kidney, and blood of WT and *Htra1* Tg mice. Htra1 is expressed at a low level but ubiquitously in all tissues analyzed. In comparison with WT, *Htra1* Tg mouse expressed 49.7-, 30-, and 3.4-fold in the retina, blood and brain, respectively. The results are expressed as the mean ± SEM. The *p* value obtained by Student's *t* test, n = 3. *B*, ELISA analysis of Htra1 expression in mouse serum. The Htra1 protein level is significantly higher in *Htra1* Tg mice than in WT mice (*p* = 0.0385, n = 16). Data are presented as the mean ± SEM. The *p* value obtained by two-tailed Mann–Whitney is indicated above the graph. *C*, the HTRA1 protein concentration in plasma or serum was determined by ELISA and it was significantly enhanced in AMD cases comparing with controls in Japanese samples (plasma, ∗∗*p* = 0.0016; serum, ∗*p* = 0.0362), Indian samples (plasma, ∗∗*p* = 0.0035; serum, ∗∗∗∗*p* < 0.0001), Australian samples (plasma, ∗∗*p* = 0.0039), samples of USA (serum, ∗*p* = 0.0293). Data are presented as the mean ± SEM. The *p* value obtained by the two-tailed Mann–Whitney test is indicated above each graph. *D*, In/del variant significantly effects HTRA1 concentration in Japanese samples (plasma, ∗∗∗*p* < 0.001; serum, ∗∗∗*p* < 0.001), Indian samples (plasma, ∗∗∗*p* < 0.001; serum, ∗∗∗*p* < 0.001), Australian samples (plasma, ∗∗∗*p* < 0.001), and samples of USA (serum, ∗∗∗*p* < 0.001) (Table S3). Data are presented as the mean ± SEM. *p* values were derived by two-way ANOVA, with Tukey's multiple comparisons test. The *p* (>0.05) value is indicated above each graph.

The CNV CAG-*Htra1* Tg mouse (17) (Fig. S4) showed increased expression in the retina, blood cells, brain, liver, and kidney compared with WT by 49.7-, 30.0-, 3.4-, 2.4-, and 2.3-fold respectively (Fig. 3*A*). The expression profile of WT mice *Htra1* mRNA was similar to that of normal human *HTRA1* mRNA (Fig. S5). The serum Htra1 protein concentration measured by ELISA was significantly higher in *CAG-Htra1* Tg mice compared with WT mice (*p* = 0.0385) (Fig. 3*B*).

### Induced concentration of blood HTRA1 protein in AMD patient with in/del

The HTRA1 concentration in serum or plasma was measured for AMD patients and controls by ELISA. A Mann–Whitney test was performed with each of the nonparametric data sets (Table S1). The average HTRA1 plasma concentration in Japanese was 150% higher in AMD compared with controls (AMD median = 0.5716 ng/ml, control median = 0.3815 ng/ml, *p* = 0.0016); 30% higher for Indian (AMD median = 1.888 ng/ml, control median = 1.451 ng/ml, *p* = 0.0035) subjects; and 32% for Australian (AMD median = 5.556 ng/ml, control median = 4.223 ng/ml, *p* = 0.0039) subjects (Fig. 3*C*). In agreement with the plasma results, serum levels of HTRA1 in AMD cases were also elevated compared with controls. The average HTRA1 serum concentration in AMD cases increased by 47% in Japanese (AMD median = 0.5722 ng/ml, control median = 0.3881 ng/ml, *p* = 0.0362), 160% in Indian (AMD median = 0.2959 ng/ml, control median = 0.1137 ng/ml, *p* < 0.0001), and 17% in American samples (AMD median = 4.323 ng/ml, control median = 3.729 ng/ml, *p* = 0.0293) subjects (Fig. 3*C*).

One of the most highly associated AMD risk variants, rs10490924, is highly associated (Lewontin's D′ of two variants is 71–98) with the in/del haplotype (Figs. S6 and S7). This finding indicates that the in/del haplotype is also likely a major risk for AMD (12, 13, 27). The frequencies of HTRA1 in/del haplotypes in both heterozygous and homozygous forms were analyzed in controls and AMD cases. The homozygous in/del variant was detected at 44.54%, 24.18%, 23.68%, and 12.20% for AMD cases and 16.95%, 10.10%, 9.68%, and 1.74% for controls respectively in samples from Japan, India, Australia, and the USA (Fig. S8). Significant association of the in/del with AMD was found in an additive genetic model in all samples by Chi-square test (Table S2).

HTRA1 blood concentration in individuals with the in/del and AMD phenotype was compared by two-way ANOVA, with Tukey's multiple comparisons test. All cases displayed significantly higher HTRA1 concentrations with the in/del haplotype in all samples (genotype *p* < 0.001). Moreover, the in/del haplotype was associated with AMD phenotype leading to an increase of HTRA1 protein concentration in plasma samples (Japan, *p* < 0.001; India, *p* = 0.001; Australia, *p* < 0.001) and serum samples (Japan, *p* < 0.001; India, *p* = 0.0437; USA, *p* = 0.0475) (Fig. 3*D* and Table S3).

### Elevation of blood HTRA1 protein concentration by aging

**Figure 4:**
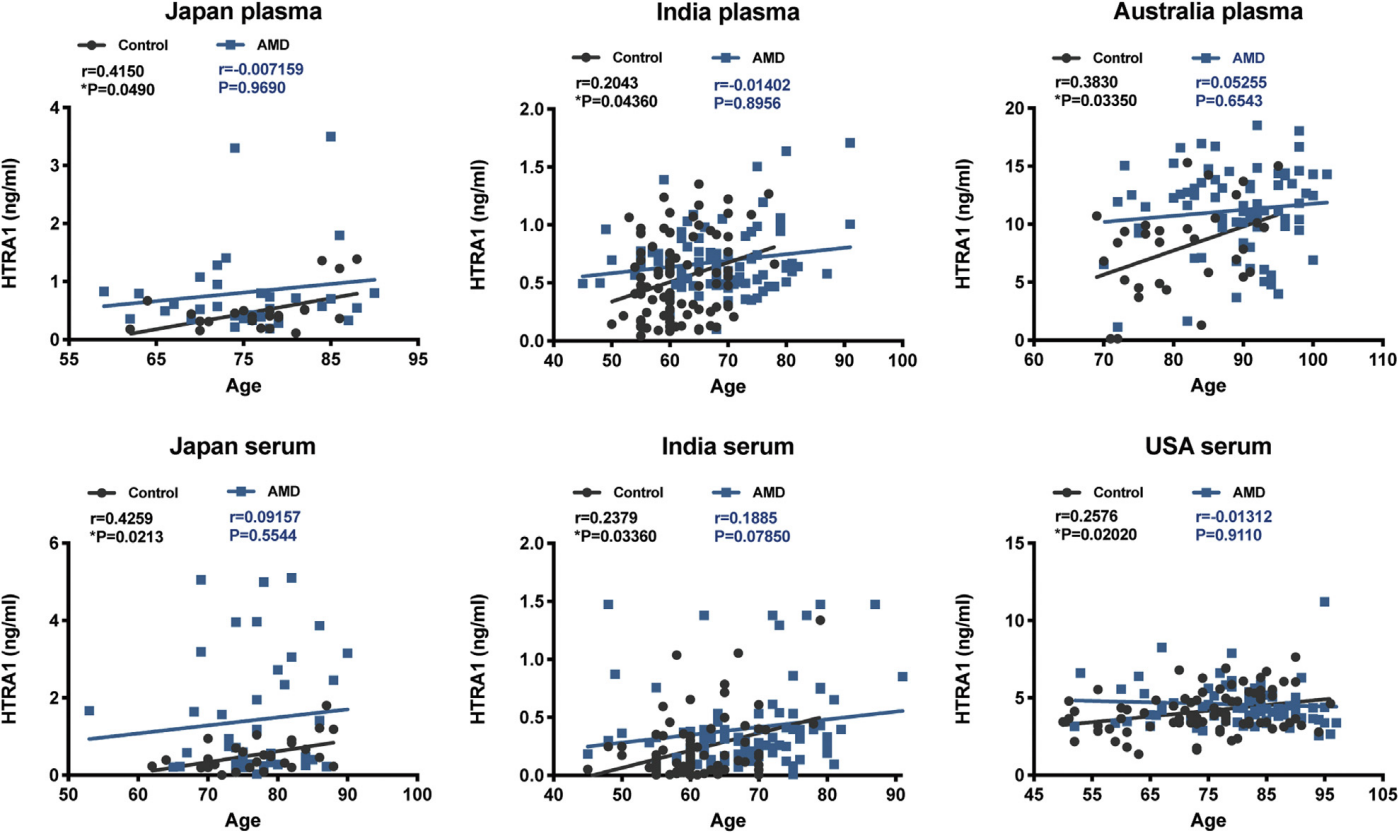
**Correlation analysis of age and blood level of HTRA1.** HTRA1 increased progressively with age in all controls. However, in AMD cases from each country, no correlation was found between blood concentration of HTRA1 and age. Data are presented as scatter plots. The linear regression equations are *solid, straight lines*. The correlation coefficient (r) and *p* (two-tailed) values were obtained using linear regression (Pearson's) analysis.

The association between HTRA1 concentration and aging was examined by linear regression analysis and Spearman's rank-order correlation. In controls there was a steady increase of HTRA1 protein in blood by age; r = 0.4150, *p* = 0.0490 in Japanese plasma; r = 0.4259, *p* = 0.0213 in Japanese serum; r = 0.2043, *p* = 0.04360 in Indian plasma; r = 0.2379, *p* = 0.03360 in Indian serum; r = 0.3830, *p* = 0.03350 in Australian plasma; r = 0.2576, *p* = 0.02020 in American serum (Fig. 4).

### Inhibition of TGF-β/ALK5/SMAD2/3 signaling by HTRA1

**Figure 5:**
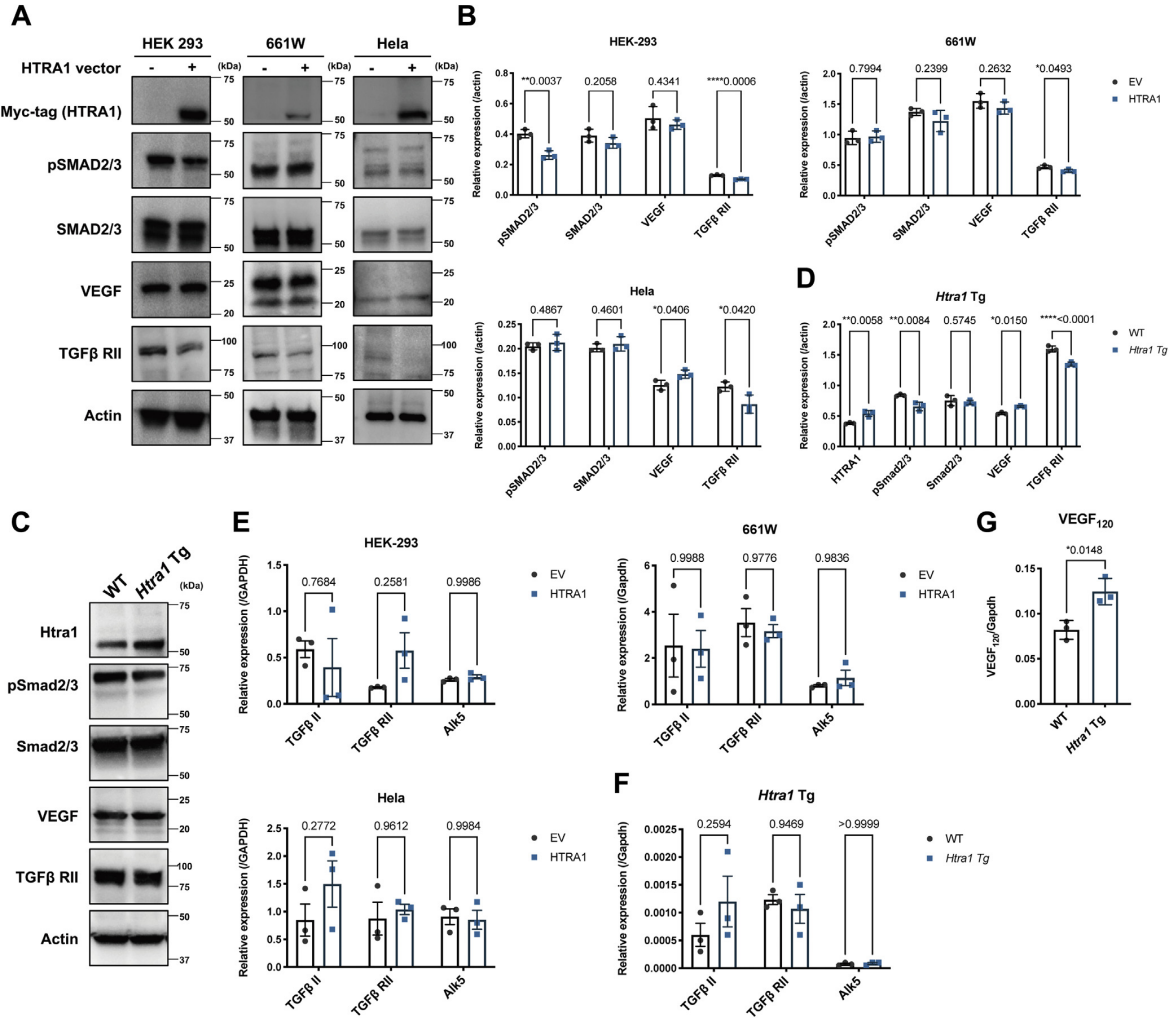
**Action of HTRA1 on TGF-β/ALK5/SMAD2/3 signaling.***A*, we assayed the effects of HTRA1 on pSMAD2/3, SMAD2/2, VEGF, and TGFβRII by transfecting pCMV-Myc-HTRA1 vector in HEK-293, 661W and HeLa cells, followed by WB. Addition of the HTRA1 expression vector significantly inhibited SAMD2/3 phosphorylation but not SMAD 2/3 expression in HEK-293 cells. HTRA1 enhanced VEGF expression in HeLa cells and cleavage TGFβRII in all three cell lines. *B*, the band intensity was analyzed by Image J software. *C*, influence of overexpressed HTRA1 in mice. Phospho-Smad2/3 and TGFβRII, but not Smad2/3, decreased in 1-year-old *Htra1* Tg mouse compared with WT mouse. VEGF enhanced in *Htra1* Tg mouse compared with WT mouse. *D*, analysis of band intensities. *E*, mRNA levels of TGF II, TGFβRII, and ALK5 in HTRA1 transfected HEK-293, 661W, and HeLa cells, respectively. qRT-PCR analysis of TGF II, TGFβRII, and Alk5 mRNA levels (*F*) and VEGF isoforms (VEGF_120_, VEGF_164_, and VEGF_188_) (*G*) in the retina of *Htra1* Tg mouse. The expression of VEGF_120_ isoform mRNA was significantly enhanced in 1-year-old *Htra1* Tg mouse compared with WT. The other two VEGF isoforms were undetectable. Throughout, the results are expressed as the mean ± SEM. The *p* value was obtained by Student's *t* test.

It is known that overexpression of HTRA1 inhibits TGF-β signaling by proteolytic degradation, which then inhibits angiogenesis through the ALK5 receptor and SMAD2/3 signaling pathway (28, 29, 30, 31). To examine HTRA1 involvement with TGF-βALK5-SMAD2/3 signaling and VEGF expression, the pCMV-Myc-HTRA1 expression vector was transfected into HEK-293, 661W and HeLa cells to observe SMAD2/3 phosphorylation and expression by WB using anti-Samd2/3 and anti-Phospho-Smad2 (ser465/467)/smad3 (ser423/425) antibodies. Increased HTRA1 expression significantly reduced SMAD2/3 phosphorylation in a dose-dependent manner without change in protein concentration in HEK-293 cells (Fig. 5, *A* and *B*). No change in VEGF expression by HTRA1 overexpression in HEK-293 and 661W cells and reduced VEGF in HeLa cells were observed (Fig. 5, *A* and *B*), excluding the possibility of direct involvement of HTRA1 in enhancing VEGF expression through the Smad2/Smad3 signaling pathways. Moreover, there was less TGF-β RII in all three kinds of cells overexpressing HTRA1 compared with controls, whereas the mRNA level remained unchanged (Fig. 5, *A*, *B*, and *E*). Our data demonstrated that HTRA1 cleaves TGF-β RII and inhibits the SMAD2/3 phosphorylation, potentially leading to a decrease in downstream TGF-β-ALK5-SMAD2/3 signaling.

Similar analysis of *CAG-Htra1* Tg mice retina revealed a significant induction of VEGF expression and TGF-β RII cleavage but reduction of Smad2/3 phosphorylation (Fig. 5, *C*, *D*, and *F*). As the VEGF gene is highly conserved with three splice variants (VEGF_120_, VEGF_164_, VEGF_188_) in mice (32), we performed a qPCR analysis to compare the expression of the three murine VEGF isoforms mRNAs in the retina. VEGF120 mRNA significantly increased in *Htra1* Tg mice compared with WT mice, but the other isoforms were undetectable (Fig. 5*G*).

## Discussion

Since the first discovery of AMD-associated susceptibility genes, namely complement factor H (*CFH*) on chromosome 1 and *ARMS2/HTRA1* on chromosome 10, increasing GWAS sample size has so far failed to uncover any other loci of higher risk (33, 34, 35). How the *ARMS2/HTRA1* locus contributes to AMD has not yet been fully resolved. We have previously suggested that enhanced expression of HTRA1 can lead to features of CNV similar to that observed in AMD and that this expression is related to the presence of an in/del and its associated transcription factors (15). We now hypothesize that the retina is not only subjected to local expression of HTRA1 but is also impacted by systemic levels of HTRA1. In turn, this increase of secreted serine protease HTRA1 from tissues outside of the eye is transported to the eye through blood circulation, thereby leading to the development of CNV.

The *Htra1* Tg mice with CNV showed statistically higher serum concentration of HtrA1 compared with WT mice. Collected plasma and serum samples from AMD patients compared with age-matched controls from Japan, India, Australia, and the USA also measured a statistically significant increase of HTRA1 protein blood concentration. These results suggest that AMD onset can potentially be predicted through the examination of the existence of the HTRA1 in/del as well as its blood concentration.

The in/del binding transcription factors GTF2I-β and δ represent two of the four human GTF2I isoforms (22, 23, 24, 25,36, 37, 38). GTF2I is a multifunctional transcription factor with the corresponding target promoter or sequences (c-fos (38), Gsc (39, 40, 41), GRP78 (42), HIV-1 LTR (43), and metabolic genes (36, 37)). Activating the *c-fos* gene with GTF2I has been associated with B-cell development (25), T-cell activation (22, 23), and mouse fibroblasts growth/proliferation (44, 45). Genetic ablation studies followed by Matrigel assays and silencing of Gtf2i in murine neonatal retina confirmed that Gtf2i plays an important role in angiogenesis and transcriptional regulation of VEGFR2 (46). Currently, the expression patterns or transcriptional functions of the GTF2I-β, γ, δ, ε isoforms are unknown (47).

Several treatments, including anti-VEGF treatment, are available for AMD. However, persistent fluid or recurrent exudation can still occur despite standardized anti-VEGF therapy in some patients (48, 49). Inhibition of the TGF-β1/3 pathway has been advocated as an optional treatment for AMD (50). In light of our results, which suggest a protective role of SMAD2/3 phosphorylation, inhibition of active HTRA1 or suppression of *HTRA1* transcription by interfering with Gtf2i β/δ binding should be advocated for AMD patients with the in/del haplotype. Further independent studies are needed to confirm this issue in order to better understand the functional consequences of activating the TGF-β2 pathway under the in/del- Gtf2i β/δ initiated upregulation of HTRA1 in different tissues outside of the eye.

We have demonstrated that elevated expression of HTRA1 in mice leads to a CNV phenotype (17). This expression is further enhanced by the role of transcription factors, and we identified the Gtf2i β/δ transcription factors in humans that modulate this process.

## Experimental procedures

### Construction of Htra1 transgenic mouse and Htra1 mRNA expression

HtrA1 Tg mice were housed in a standard animal maintenance facility under a 12-h light: 12-h dark cycle (17). All procedures for animal experiments were performed in accordance with the animal experiment guidelines of the National Hospital Organization. All experiments were approved by the Experimental Animal Committee of the National Hospital Organization Tokyo Medical Center. Total RNA from different tissues was purified using the RNeasy Mini Kit (Qiagen) according to the manufacturer's instructions. The yield was determined by NanoDrop ND-1000 spectrophotometer (NanoDrop Technologies). For quantitative reverse transcription-PCR (RT-PCR) analysis, cDNA was synthesized from 400 ng of total RNA in a 20 μl reaction mixture using High-Capacity cDNA Reverse Transcription Kit (Applied BioSystems) according to the manufacturer's instructions. Real-time quantitative RT-PCR was performed with synthesized cDNA using SsoAdvanced Universal SYBR Green Supermix (BioRad) by ABI STEP-One Real-time PCR system (Life Technologies) and primers for genes of interest. Experiments were performed three times for each sample and then compared respectively by Students' *t* test.

### Optical coherence tomography observation of CNV mouse

Retinal section imaging was performed using optical coherence tomography (OCT) on 12-month-old *HtrA1* Tg mice under ketamine (Daiichi Sankyo) injection anesthesia at 0.002 ml/g body weight into their abdominal cavities. Pupils were dilated with 5 mg/ml Tropicamide (Santen Pharmaceutical). Fundus examinations and OCT studies were performed using Micron III (Phoenix Research Products) and Spectralis HRA + OCT (Heidelberg Engineering), respectively.

### Histology and immunohistochemistry

After fixing with 5% formaldehyde, the eyes were embedded in paraffin and sectioned at 5-μm thickness. Following deparaffinization and rehydration, sections were hematoxylin and eosin (H&E) stained. The images were collected by Nikon Eclipse light microscope (Nikon Corporation). For immunohistochemistry, the sections were treated with Target Retrieval Solution (DakoCytomation) at 120 °C for 10 min. After blocking, they were incubated with anti-CD31 antibody (1:50; Santa Cruz Biotechnology) overnight at 4 °C and then were incubated with Alexa Fluor 488 conjugated rabbit anti-mouse IgG (1:500; Life Technologies) and 4′6-diamidino-2-phenylindole (DAPI). Slides were mounted with Ultramount Aqueous Permanent Mounting Medium (DakoCytomation) and observed with a confocal fluorescence laser microscope (LSM 700).

### Study subjects

Unrelated AMD patients and age- and gender-matched control subjects were recruited at Tokyo Medical Center, Japan (229 patients and 236 controls), at L V Prasad Eye Institute, Hyderabad, India (91 patients and 99 controls), at the Centre for Eye Research Australia, University of Melbourne, Australia (76 patients and 31 controls), and at Moran Eye Center, University of Utah, Salk Lake City, USA (74 patients and 81 controls), respectively (Table 2). All study subjects were given complete ophthalmoscopic examinations. The control subjects did not have a family history of AMD, signs of AMD, or any other major eye diseases except early senile cataracts and low myopia. Their fundus was normal; there were no drusen, abnormal RPE change, and the foveal reflex was present in all control eyes. The study was in accordance with the tenets of the Declaration of Helsinki and informed consent was obtained from all participants.

**Table 2:** Demographic characteristics of study subjects

Sample	AMD	Control	*p* Value
Japan			
Subjects	229	236	
Female, n (%)	57 (24.8%)	161 (67.9%)	
Age^a^ range (years)	51–92	50–90	
Mean age ± SD (years)	74.8 ± 8.8	75.4 ± 7.3	0.63
Japan blood sample			
Subjects	44	29	
Female, n (%)	11 (25%)	17 (58.6%)	
Age range (years)	53–90	62–88	
Mean age ± SD (years)	76.7 ± 7.0	76.9 ± 7.1	0.91
India			
Subjects	91	99	
Female, n (%)	25 (27.4%)	52 (52.5%)	
Age range (years)	44–91	45–91	
Mean age ± SD (years)	68.0 ± 8.8	68.0 ± 8.8	0.97
Australia			
Subjects	76	31	
Female, n (%)	46 (60.5%)	18 (58.1%)	
Age range (years)	70–102	69–95	
Mean age ± SD (years)	88.9 ± 7.7	85.5 ± 7.5	0.11
USA			
Subjects	82	115	
Female, n (%)	34 (45.9%)	30 (40.5%)	
Age range (years)	52–97	50–96	
Mean age ± SD (years)	80.4 ± 10.3	79.3 ± 11.1	0.06
Combined			
Subjects	470	447	
Female, n (%)	162 (34.5%)	261 (58.3%)	
Age range (years)	45–102	45–97	
Mean age ± SD (years)	75.6 ± 11.1	75.12 ± 9.2	0.52

SD, standard deviation.

*p* Value of Indian samples was obtained from unpaired *t* test (two-tailed); the others were obtained from Mann–Whitney test (two-tailed).

a Age of presentation.

### Cell culture, transfection, and nuclear protein extraction

661W, COS-7, and HEK-293 cells were grown in DMEM supplemented with 10% fetal calf serum (FCS). hiPSCs were obtained and cultured as previously described (15). Briefly, 4.5 × 10^5^ human iPS cells were expanded in COAT-1 (Cellartis def-cs 500, TaKaRa) coated six-well plates in Basal medium (Cellartis def-cs 500, TaKaRa). The culture supernatant was collected for ELISA after 48 h. ViaFect transfection reagent (Promega) and opti-MEM were used to transfect the plasmids in accordance with the supplier's protocol. The amounts of plasmids and oligonucleotides, cell numbers, and plates used were as follows: 2 ng of plasmids and 12 ul transfection reagent into 4 × 10^5^ cells using 6-well plate. Nuclear protein was extracted using the CelLytic NuCLEAR Extraction Kit (Sigma) following the manufacture's protocol. The protein concentration was determined with the BCA protein assay kit (Thermo Scientific).

### Genotyping of *ARMS2* in/del haplotype

Genotyping was done as described previously (48). Briefly, genomic DNA from blood samples of the study subjects was extracted (Magtration System 8Lx QIAamp; DNA Kit; Precision System Science Co., Ltd, Tokyo, Japan) according to the supplier's instructions. The genotype of in/del (EU427539, chr:124206811–124207253) in ARMS2 (ENSG00000228258) was determined by PCR (forward primer: 5′- ACATATCTCCTTAAAAGCCAACTG -3′, reverse primer: 5′- ATCCATCCCTACTCACCCATTA -3′) using PrimeSTAR HS DNA Polymerase (Takara) and direct sequencing (BigDye Terminator v3.1 Cycle Sequencing Kit; Thermo Fisher Scientific) on a DNA sequencer (ABI 3130; Applied Biosystems).

### Plasma collection

Whole-blood samples were collected by venipuncture into heparin-coated glass vacutainer tubes. All blood samples were mixed by inverting several times to hemolyze. After centrifuging for 10 min at 4 °C (2000*g*), plasma was transferred to Cryo tube (Nunc Cat. No. 375418) and frozen at –80 °C until analysis by ELISA.

### Serum samples collection and extraction

Single-point blood samples were collected by venipuncture into uncoated glass vacutainer tubes. All blood samples were allowed to clot at room temperature for 2 h and then centrifuged (1300 ) at 4 °C for 10 min. The serum was transferred and frozen at –80 °C until analysis by ELISA.

### Assessment of HTRA1 blood level

The HTRA1 concentration was measured by ELISA (Human HtrA1 serine peptidase 1 ELISA kit, MBS454847, MyBioSource; Mouse Serine Protease HTRA1 ELISA Kit, MBS2882375, MyBioSource). It was performed according to the manufacture's instruction. For human blood sample measurement, 12 μl of samples was diluted to 120 μl using sample diluent buffer just prior to the assay. The cell culture supernatant was directly applied without dilution.

### Quantitative real-time PCR

For mice, total RNA from different tissues was purified using RNeasy Mini Kit (Qiagen) according to the manufacturer's instructions. The yield was determined by NanoDrop ND-1000 spectrophotometer (NanoDrop Technologies). For quantitative reverse transcription-PCR (RT-PCR) analysis, cDNA was synthesized from 400 ng of total RNA in a 20 μl reaction mixture using High-Capacity cDNA Reverse Transcription Kit (Applied BioSystems) according to the manufacturer's instructions. Real-time quantitative RT-PCR was performed with synthesized cDNA using SsoAdvanced Universal SYBR Green Supermix (BioRad) by ABI STEP-One Real-time PCR system (Life Technologies) and primers for genes of interest. By the same token, for humans, real-time quantitative RT-PCR was performed with cDNA library (Clontech) using SsoAdvanced Universal SYBR Green Supermix (BioRad). GAPDH was used as the normalizing control. Primer sequences are listed in Table S4.

### Immunocytochemistry of cultured cells

Transfected cells were fixed in 4% paraformaldehyde for 15 min and permeabilized in 0.1% Triton X-100 for 5 min. Samples were washed three times with 1% PBST, followed by blocking with protein block serum-free (Dako) for 1 h and incubation with ANTI-FLAG M2 (1:500; F1804, SIGMA)/anti-firefly luciferase antibody (1:200; ab21176, Abcam) overnight at 4 °C. Following three washes with PBST, the samples were incubated with Alexa Fluor 568-conjugated goat anti-mouse IgG antibodies (1:500; Invitrogen)/Alexa Fluor 488-conjugated goat anti-rabbit IgG antibodies (1:500; Invitrogen) and DAPI (1:500; Dojindo) for 1 hour at RT to visualize the antigens and nuclei. These were mounted with Ultramount aqueous permanent mounting medium (DakoCytomation) and visualized under a confocal fluorescent microscope (LSM700, Zeiss).

### Western blotting

WB was performed on cell samples homogenized in an appropriate volume of ice-cold TNE buffer (10 mM Tris-HCl, 100 mM NaCl, 10 mM EDTA, 0.1% (v/v) Nonidet P-40, pH 7.4) containing protease and phosphatase inhibitor (Roche Applied science). The homogenate was kept on ice for 30 min and then centrifuged (13,000*g*, 5 minutes, 4 °C). The supernatant was removed and the protein concentrations were determined by Pierce BCA protein assay (Thermo Scientific). Equal amounts of the supernatant were separated on 7.5% SDS-PAGE and blotted onto PVDF membranes (Trans-Blot Turbo, Bio-Rad). Blots were probed with primary antibodies, respectively. Every blot was also probed with a polyclonal antibody for actin or lamin A/C to ensure equal protein loading. Detection of protein was achieved with SuperSignal West Femto maximum sensitivity substrate (Thermo) using the Bio-Rad system (ChemiDoc XRS+).

### Primary antibodies

The primary antibodies used in WB included: TFII-I antibody (1:1000; CST; #4562), anti-hnRNP K antibody (1:10,000; Abcam; ab52600), EF-1r polyclonal antibody (1:1000; SAB; #40863), Lamin A/C (4c11) antibody (1:2000; CST; #4777); anti-actin (1/1000; Millipore; #MAB1501), ANTI-FLAG M2 (1:1000; SIGMA; F1804), anti-firefly luciferase antibody (1:1000; Abcam, ab21176), Samd2/3(D7F7) (1:1000; CST; #8685), Phospho-Samd2 (ser465/467)/smad3 (ser423/425) (D27F4) (1:1000; CST; #8828), anti-VEGF (1:1000; Abcam; ab46154), anti-HTRA1(1:500; R&D, MAB2916), anti-TGFβ RII(D-2) (1:300S; CB; sc-17799).

### Secondary antibodies

The secondary antibodies used in WB included: Donkey Anti-Goat IgG H&L (HRP) (1/10,000; Abcam; ab97110), Goat anti-rat IgG-HRP (1/10,000; SCB; sc-2006).

### Plasmid construction and oligonucleotide preparation

The pCMV donor vectors carrying Gtf2i β/δ or HTRA1 were constructed by PCR and TA-cloning with TArget Clone (Toyobo). For the construction of the pCMV-flag Gtf2i or pCMV-Myc HTRA1 vector, the flag sequence was added by PCR and In-Fusion cloning (Takara). The primers are listed in Table S4.

### Electrophoretic mobility shift assay (EMSA)

EMSA was carried out using a LightShift Chemiluminescent EMSA kit (Thermo Scientific) as described previously. Briefly, the double-stranded 5′-biotinylated DNAs were synthesized as Table S5. After incubating with probes for 20 min, the nuclear extracts from 661W were separated by 7% EMSA gel and transferred onto Biodyne B precut modified nylon membranes (Thermo Scientific). The membranes were cross-linked in a UV transilluminator for 15 min and then were incubated with blocking buffer and streptavidin-horseradish peroxidase conjugates. Bound conjugates were detected using a molecular imager (Chemi Doc XRS+, Bio-Rad).

### Isolation of in/del binding transcription factor(s) and identification by proteomic analysis using LC-MS/MS

Isolation of in/del binding transcription factor(s) was performed by EMSA using the nuclear extract from 661W mouse cell line and 40 bp double-strand DNA probes designed to overlap the 100 bp in/del sequence. 5′-end DNA probes attached to biotin magnetic beads were used to pull out in/del binding transcription factors and further identified by liquid chromatography–mass spectrometer (LC-MS/MS) as described previously (Fig. 1, *A* and *B*) (15). Briefly, the nuclear extract of 661W cells (50 μg) was combined with 100 pmol of biotin-labeled, double-stranded DNA probes in DNAP buffer (20 mm HEPES, 80 mm KCl, 1 mm MgCl, 0.2 mm EDTA, 10% glycerol, 0.1% Triton X-100, 0.5 mm DTT). The protein/probe mixture was incubated at 4 °C for 30 min before 50 ml of Dynabeads M280 streptavidin (Invitrogen) was added. The mixture was incubated at 4 °C for 30 min. The beads-probes-protein complex was then washed with 500 μl of DNAP buffer three times for 30 min at 4 °C. The washed complexes were mixed with 30 μl of SDS-PAGE sample buffer (Bio-Rad) and were boiled for 5 min at 100 °C. The boiled samples were quenched on ice for 5 min before being separated by 7.5% SDS-PAGE. Bands of interest were cut out of the gel and were processed for in-gel digestion for further LC-MS/MS analysis. A Thermo LTQ system (Thermo Scientific) and Scaffold 4 data analysis software (Matrix Science) were used.

### Statistical analyses

All data analysis was performed by statistical software (Prism 8; GraphPad). To compare allelic or genotypic frequencies, Fisher's exact test was used in three different models (allelic, dominant, and recessive forms) of each case group with controls. In the allelic model, the allelic frequencies were compared between cases and controls using a 2 × 2 contingency table; in the dominant model, the frequencies of the homozygote for the nonrisk allele were compared using a 2 × 2 contingency table, and in the recessive model, the frequencies of the homozygote for the risk allele were compared using a 2 × 2 contingency table. For the additive model, Chi-square test was performed. The frequencies of the three genotypes were compared using a 2 × 3 contingency table and association analysis was performed with the use of Chi-square trend test. After statistical analysis in four different models (allelic, recessive, dominant, and additive model) and trend test, the minimum *p* value was obtained from the lowest *p* value of these tests. The minimum *p* values were used to test whether they were lower than the significance levels. Odds ratios (ORs) with 95% confidence intervals (CIs) were estimated for the effects of risk allele and also for both the dominant and recessive forms of the genotypes. The distribution of HTRA1 concentration was estimated by D'Agostino Pearson omnibus normality test, Shapiro–Wilk normality test, and Kolmogorov–Smirnov test with Dallal–Wilkinson–Lilliefors *p* value. The difference of HTRA1 concentration was compared using nonparametric Mann–Whitney test. Two-tailed probabilities of less than 0.05 were considered significant. Interaction of genotype effects on HTRA1 concentration was evaluated by two-way ANOVA, and it was employed at a significance level of 0.05. The significance of correlations between HATRA1 concentration and age in the case control was tested using Pearson's correlation coefficient. Pairwise SNP LD values were calculated from the genotype data using Haploview.jar (13).

## Data availability

The data used to support the findings of this study are available from the corresponding author upon request.

## Supporting information

This article contains supporting information.

## Conflict interest

Takeshi Iwata is funded by Daiichi Sankyo, Inc. Robyn H. Guymer is on the advisory boards of Bayer, Novartis, Roche Genentech, and Apellis. The remaining authors declare no competing interest.
