# Supplementary material for: Binding of Gtf2i-β/δ transcription factors to the ARMS2 gene leads to increased circulating HTRA1 in AMD patients and in vitro
Source: J Biol Chem. 2021 Feb 24;296:100456. doi: 10.1016/j.jbc.2021.100456 (PMC8039566; doi:10.1016/j.jbc.2021.100456)

Identification of Gtf2i-β/δ transcription binding factors to the *ARMS2* insertion/deletion leads to systemic upregulation of HTRA1 levels in blood resulting in AMD

Yang Pan [1], Daisuke Iejima [1], Mao Nakayama [1], Akiko Suga [1], Toru Noda [2], Inderjeet Kaur [3], Taraprasad Das [3], Subhabrata Chakrabarti [3], Robyn H. Guymer [4,6], Margaret M. DeAngelis [5], Megumi Yamamoto [1, 7], Paul N. Baird [6]*, Takeshi Iwata [1]*#

Supplementary Figure 1

a

Matrix ID	Name	Score	Relative score	Sequence ID	Start	End	Strand	Predicted sequence
**MA0099.3**	**FOS::JUN**	**4.82266**	**0.7913389**	**EU427539.1**	**9**	**18**	**-**	**ATTAATAACC**
**MA0099.3**	**FOS::JUN**	**2.76155**	**0.75145164**	**EU427539.1**	**10**	**19**	**+**	**GTTATTAATT**
**MA0099.3**	**FOS::JUN**	**-0.178367**	**0.6945573**	**EU427539.1**	**17**	**26**	**+**	**ATTAATTAAC**
**MA0099.3**	**FOS::JUN**	**-0.214463**	**0.69385875**	**EU427539.1**	**36**	**45**	**+**	**ATTATTTAGT**
MA0099.3	FOS::JUN	-1.43122	0.67031168	EU427539.1	13	22	+	ATTAATTAAT
MA0099.3	FOS::JUN	-1.43122	0.67031168	EU427539.1	13	22	-	ATTAATTAAT
MA0099.3	FOS::JUN	-1.48229	0.66932326	EU427539.1	35	44	-	CTAAATAATT
MA0099.3	FOS::JUN	-1.86473	0.66192231	EU427539.1	49	58	-	GTTAATTAAA
MA0099.3	FOS::JUN	-2.05962	0.65815065	EU427539.1	21	30	-	TTTAGTTAAT
MA0099.3	FOS::JUN	-2.05962	0.65815065	EU427539.1	40	49	+	TTTAGTTAAT
MA0099.3	FOS::JUN	-2.05962	0.65815065	EU427539.1	53	62	-	TTTAGTTAAT
MA0099.3	FOS::JUN	-2.52077	0.64922623	EU427539.1	12	21	-	TTAATTAATA
MA0099.3	FOS::JUN	-2.61048	0.64749029	EU427539.1	17	26	-	GTTAATTAAT
MA0099.3	FOS::JUN	-3.07328	0.63853402	EU427539.1	65	74	-	CTCACCCATT
MA0099.3	FOS::JUN	-3.26652	0.63479421	EU427539.1	14	23	+	TTAATTAATT
MA0099.3	FOS::JUN	-3.26652	0.63479421	EU427539.1	16	25	-	TTAATTAATT
MA0099.3	FOS::JUN	-3.82483	0.62398961	EU427539.1	49	58	+	TTTAATTAAC
MA0099.3	FOS::JUN	-4.11021	0.61846697	EU427539.1	18	27	+	TTAATTAACT
MA0099.3	FOS::JUN	-4.11021	0.61846697	EU427539.1	50	59	+	TTAATTAACT
MA0099.3	FOS::JUN	-4.46461	0.61160853	EU427539.1	32	41	+	TTAAATTATT
MA0099.3	FOS::JUN	-4.58575	0.60926401	EU427539.1	54	63	+	TTAACTAAAC
MA0099.3	FOS::JUN	-4.78834	0.60534355	EU427539.1	58	67	-	ATTAGTTTAG

b

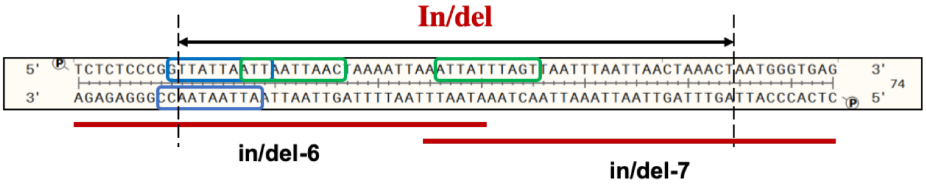

Supplementary Figure 1. Linking *c-fos* transcription factor to in/del-6 and 7. GTF2I binding site prediction by JASPAR (a) relative profile score threshold is 60% and their positions in the indel regions of ARMS2 genes (b). The binding sequence with the high relative score (blue) is located in the probe of in/del-6, and the sequence of lower score find in in/del-6 and in/del-7(green).

Supplementary Figure 2

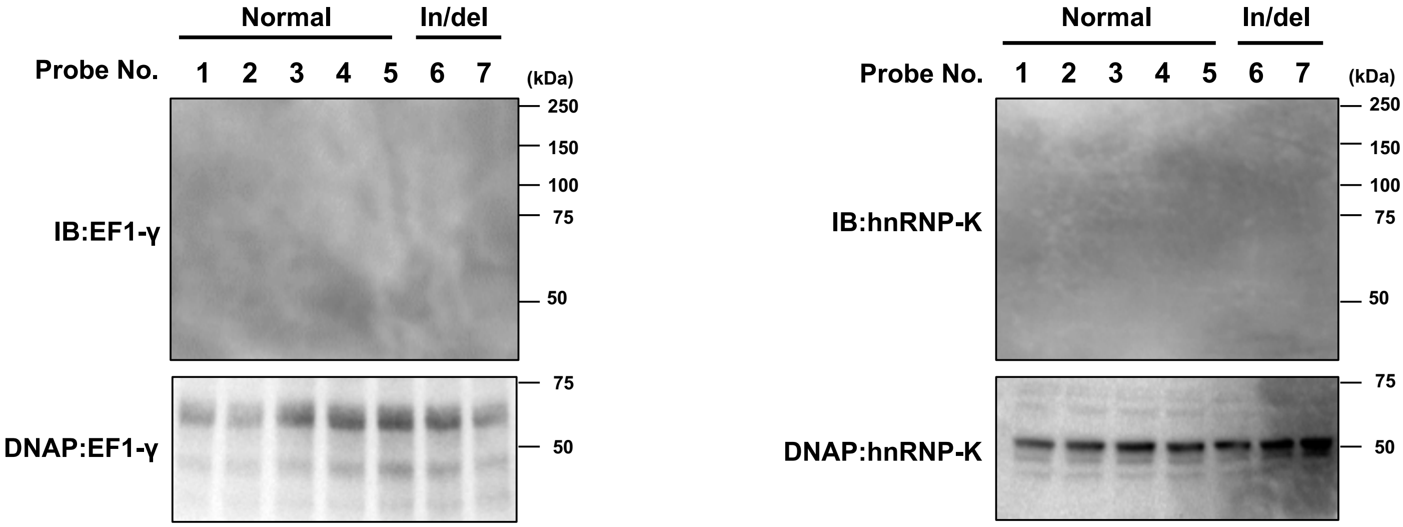

Supplementary Figure 2. EF1/hnRNP-K with DNA probes binding test. Neither of EF1 or hnRNP-K can be detected in any of DNA-protein precipitation by WB. DNAP of all probes was detected by anti-EF1 or anti-hnRNP-K antibody. Detection of Lamin A/C was used as an internal control (Figure 1c).

Supplementary Figure 3

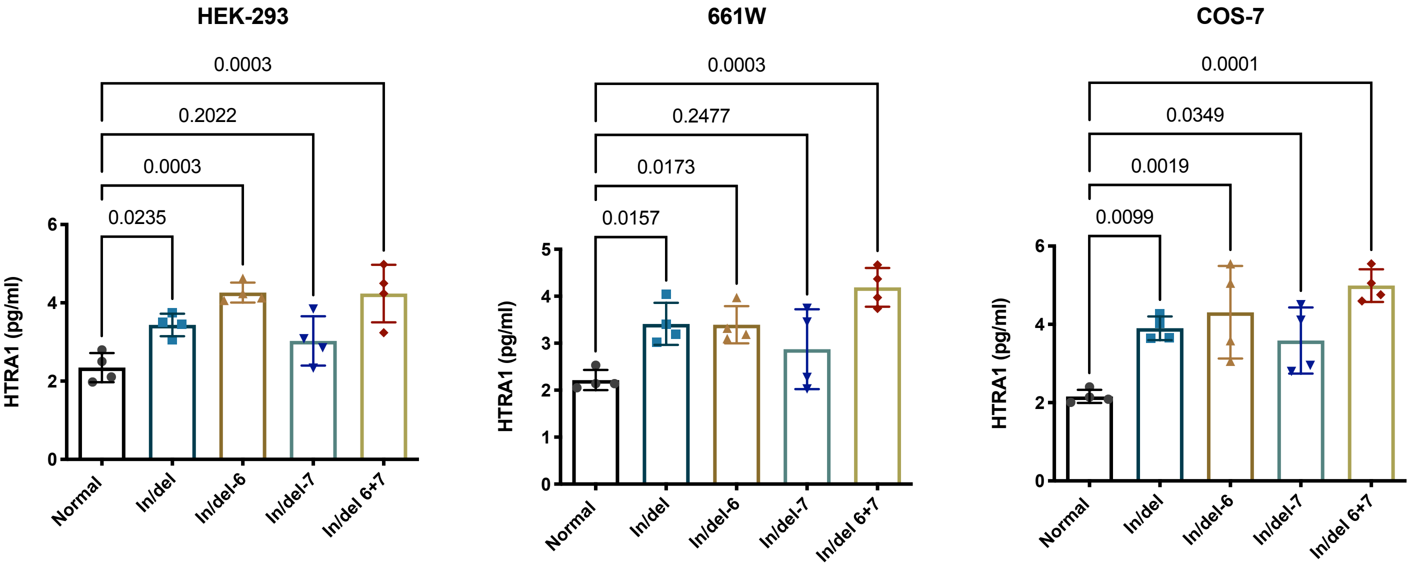

Supplementary Figure 3. Secretion of HTRA1 in vitro. Five kinds of expression vectors (normal, in/del, indel-6, indel-7 and co-indel-6/7) were transfected in HEK-293, 661W and COS-7 cells, respectively, and followed by ELISA. In/del and in/del-6 scientifically enhanced HTRA1 secretin in all cell lines. The results are expressed as the mean ± SEM. The P value was obtained by Student's t-test.

Supplementary Figure 4

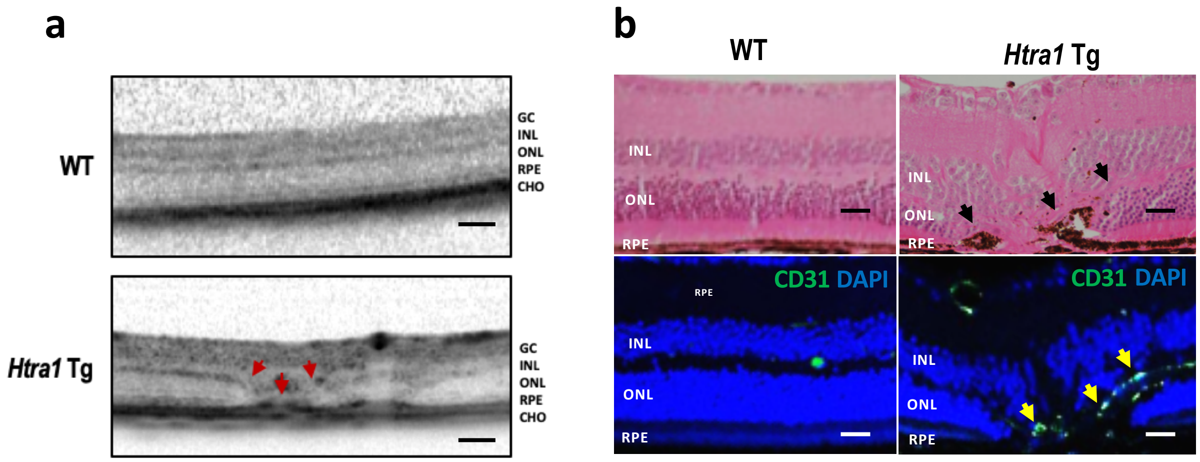

Supplementary Figure 4. The *Htra* 1 Tg mice developed CNV. (a) The CNV was observed in the choroid and retina by OCT (red arrowheads; scale bar: 200 μm) in one-year-old Htra1 Tg mice. (b) The H&E staining of Htra1 Tg mouse showed radial CNV spreading from the choroid through the RPE into the retina (black arrowheads; ×40 magnification; scale bars: 20 μm). The endothelial cell marker CD31 was stained (green) in a radial CNV (yellow arrowheads; scale bars: 20 μm). Nuclei were stained with DAPI (blue).

Supplementary Figure 5

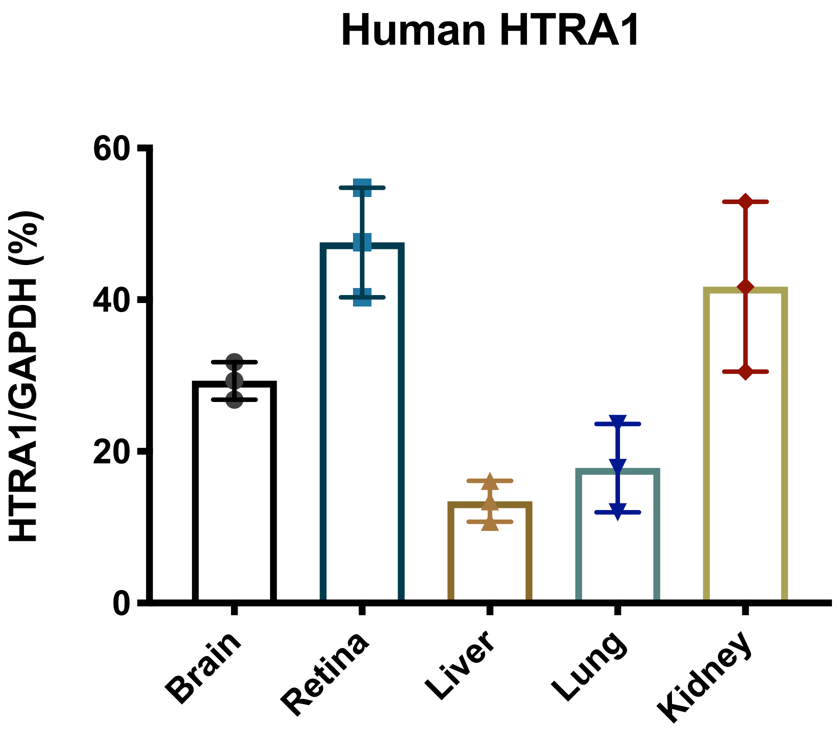

Supplementary Figure 5. HTRA1 mRNA expression in human. Quantitative real-time PCR determination of HTRA1 mRNA levels from cDNA library of human brain, retina, liver, lung and kidney. HTRA1 is ubiquitously expressed in all tissues and examined at very low levels. The results are expressed as the mean ± SEM.

Supplementary Figure 6

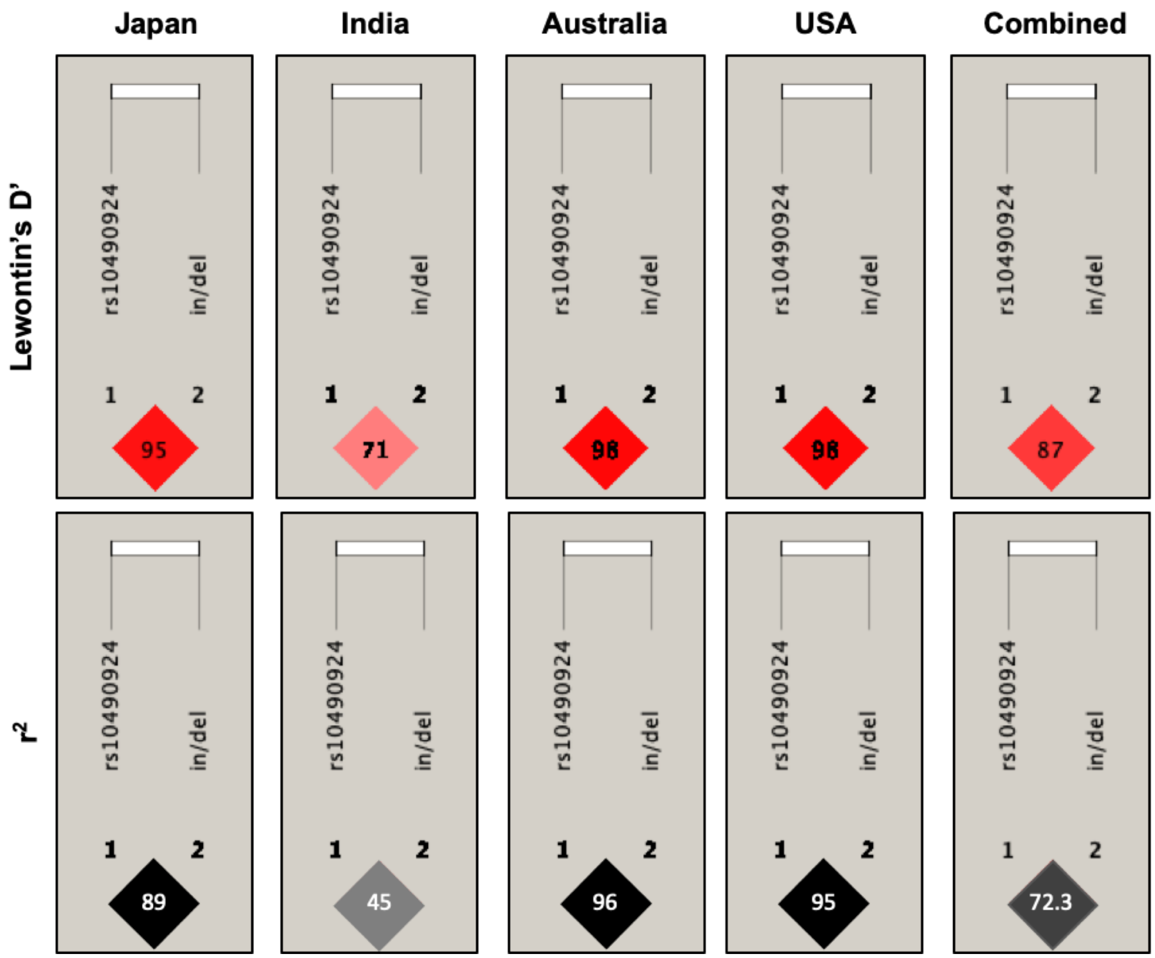

Supplementary Figure 6. LD plots of the two variants (rs10490924 and in/del) in Japan, India, Australia, USA and combined samples. The D' and r2 values between two SNPs are indicated inside the quadrants for each dataset, respectively.

Supplementary Figure 7

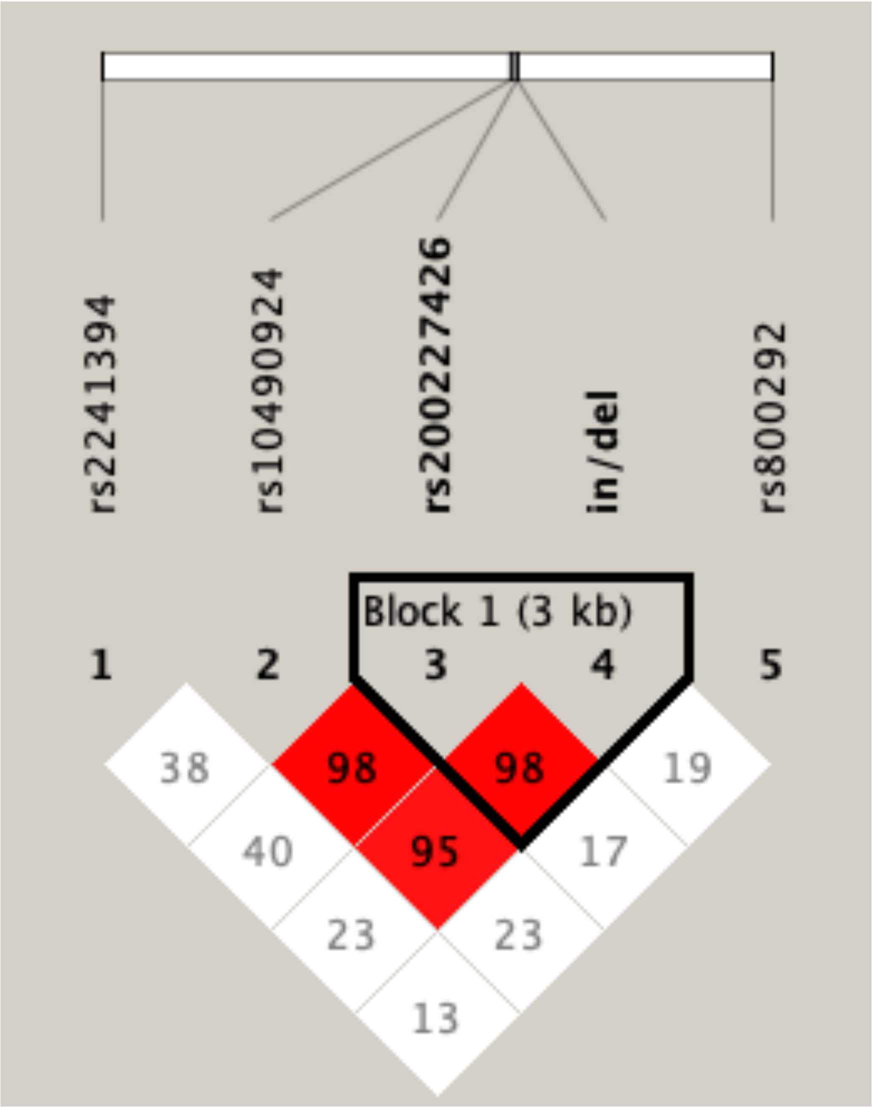

Supplementary Figure 7. Linkage disequilibrium plots of the five variants (rs2241394, rs10490924, rs200227426, rs800292 and in/del) in Japan samples. The D' values between each SNPs are indicated inside the quadrants for the Japan samples.

Supplementary Figure 8

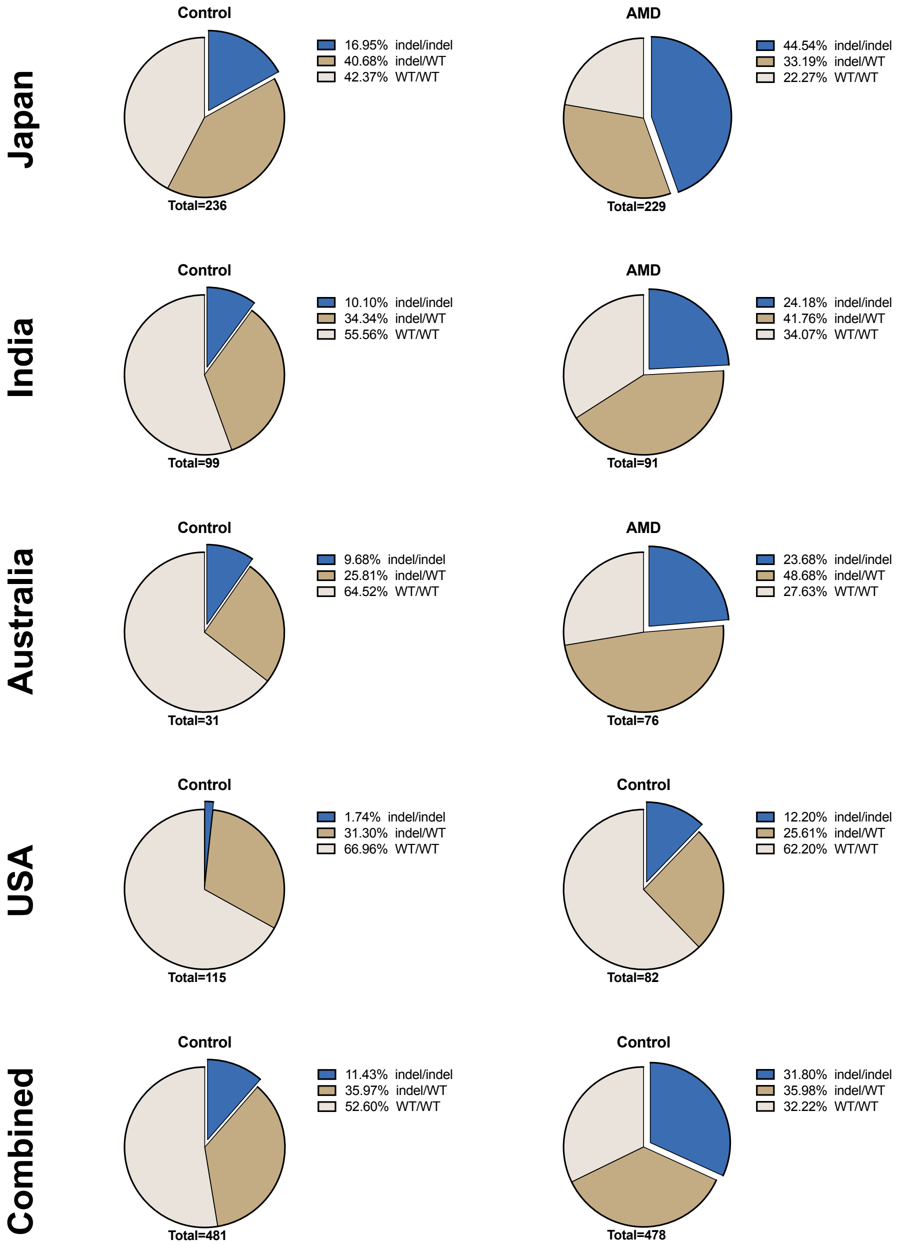

Supplementary Figure 8. Genotypic distribution of in/del variant in controls and AMD patients. The in/del fraction of total is computed for the Japan, India, Australia, USA and combined samples, respectively. The frequency of in/del is significantly higher in AMD samples comparing with controls.

Supplementary Figure 9

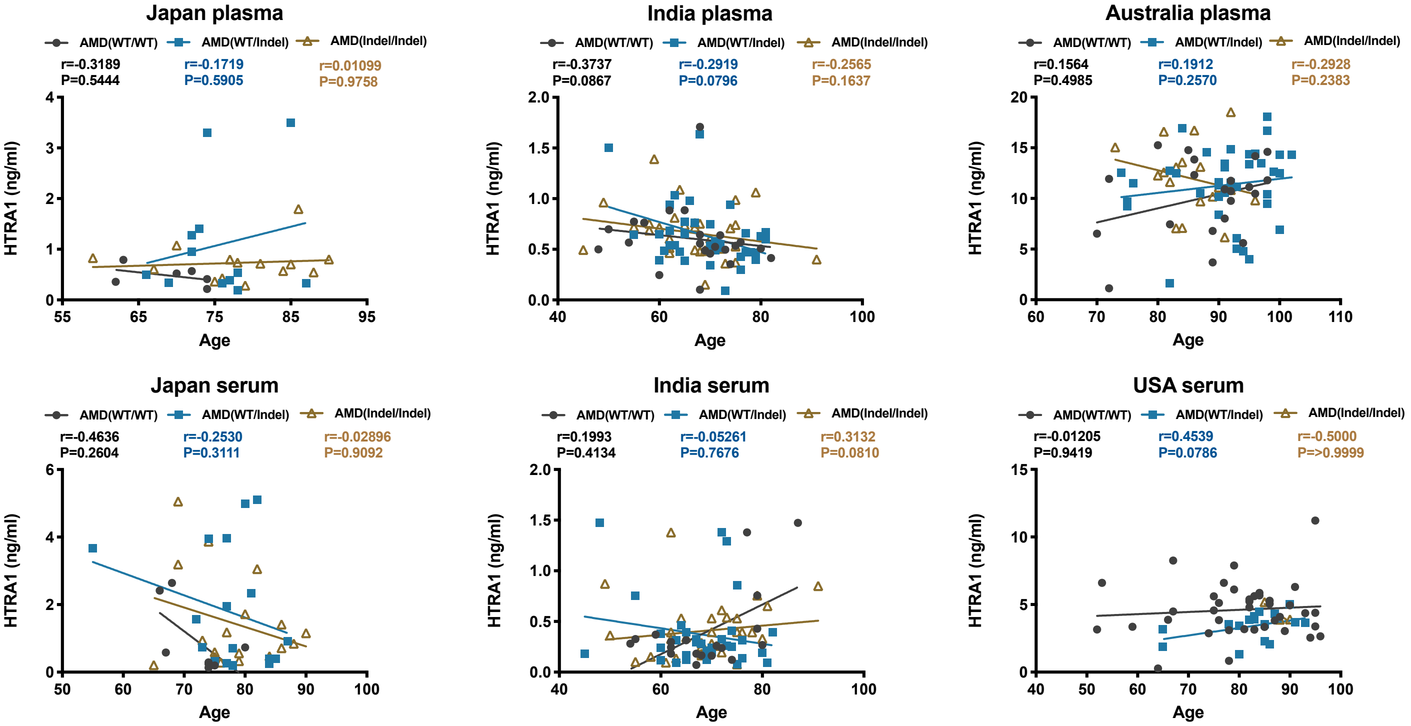

Supplementary Figure 9. Correlation analysis of age and blood level of HTRA1 in AMD patients. In all indel genotype AMD cases from each country, no correlation was found between blood concentration of HTRA1 and age. Data are presented as scatter plots. The linear regression equations are solid, straight lines. The correlation coefficient (r) and *P* (two tailed) values were obtained using linear regression (Pearson's) analysis.

Supplementary Table 3

Supplementary Table 4. Results from a two-way ANOVA showing main effects and interaction of the genotype effects to HTRA1 concentration in blood samples.

		Source of Variation					ANOVA table					
			% of total variation	P value	P value summary	Significant		SS	DF	MS	F (DFn, DFd)	P value	Number of missing values
Japan	Plasma	Interaction	11.93	< 0.0001	****	Yes	Interaction	1.40E+06	2	699378	F (2, 67) = 48.36	P < 0.0001	35
		Row Factor	16.24	< 0.0001	****	Yes	Genotype	1.90E+06	2	952164	F (2, 67) = 65.84	P < 0.0001	
		Column Factor	63.57	< 0.0001	****	Yes	Phenotype	7.46E+06	1	7.46E+06	F (1, 67) = 515.5	P < 0.0001	
							Residual	968990	67	14463			
	Serum	Interaction	11.93	< 0.0001	****	Yes	Interaction	1.40E+06	2	699378	F (2, 67) = 48.36	P < 0.0001	35
		Row Factor	16.24	< 0.0001	****	Yes	Genotype	1.90E+06	2	952164	F (2, 67) = 65.84	P < 0.0001	
		Column Factor	63.57	< 0.0001	****	Yes	Phenotype	7.46E+06	1	7.46E+06	F (1, 67) = 515.5	P < 0.0001	
							Residual	968990	67	14463			
India	Plasma	Interaction	2.158	0.001	***	Yes	Interaction	22551	2	11276	F (2, 161) = 7.220	P = 0.0010	103
		Row Factor	17.74	< 0.0001	****	Yes	Genotype	185354	2	92677	F (2, 161) = 59.34	P < 0.0001	
		Column Factor	56.04	< 0.0001	****	Yes	Phenotype	585637	1	585637	F (1, 161) = 375.0	P < 0.0001	
							Residual	251452	161	1562			
	Serum	Interaction	1.363	0.0437	*	Yes	Interaction	773.7	2	386.9	F (2, 161) = 3.192	P = 0.0437	103
		Row Factor	5.695	< 0.0001	****	Yes	Genotype	3233	2	1616	F (2, 161) = 13.34	P < 0.0001	
		Column Factor	58.56	< 0.0001	****	Yes	Phenotype	33240	1	33240	F (1, 161) = 274.3	P < 0.0001	
							Residual	19513	161	121.2			
Australia	Plasma	Interaction	1.627	< 0.0001	****	Yes	Interaction	3.779	2	1.89	F (2, 101) = 13.95	P < 0.0001	115
		Row Factor	16.08	< 0.0001	****	Yes	Genotype	37.34	2	18.67	F (2, 101) = 137.9	P < 0.0001	
		Column Factor	76.41	< 0.0001	****	Yes	Phenotype	177.5	1	177.5	F (1, 101) = 1311	P < 0.0001	
							Residual	13.68	101	0.1354			
USA	Serum	Interaction	0.5205	0.0475	*	Yes	Interaction	0.2049	2	0.1025	F (2, 149) = 3.111	P = 0.0475	139
		Row Factor	85.03	< 0.0001	****	Yes	Genotype	33.48	2	16.74	F (2, 149) = 508.2	P < 0.0001	
		Column Factor	1.984	< 0.0001	****	Yes	Phenotype	0.7813	1	0.7813	F (1, 149) = 23.72	P < 0.0001	
							Residual	4.907	149	0.03294			

Supplementary Table 4

Supplementary Table 4. List of primers used in the study

	Primer name	Sequence
Quantitative real-time PCR	mouse HtrA1 forward	GATTGACCACCAGGGAAAGC
	mouse HtrA1 reverse	TTCCGAAGTCCCAGCTCTTT
	mouse GAPDH forward	GTTGTCTCCTGCGACTTCA
	mouse GAPDH reverse	GGTGGTCCAGGGTTTCTTA
	human HTRA1 forward	GTGGATGACG GATGAGGACT
	human HTRA1 reverse	AGGATACAGAAGGGCAGAGC
	mouse VEGF120 forward	GCCAGCACATAGGAGAGATGAGC
	mouse VEGF120 reverse	CGGCTTGTCACATTTTTCTGG
	mouse VEGF164 forward	GCCAGCACATAGGAGAGATGAGC
	mouse VEGF164 reverse	CAAGGCTCACAGTGATTTTCTGG
	mouse VEGF188 forward	GCCAGCACATAGGAGAGATGAGC
	mouse VEGF188 reverse	AACAAGGCTCACAGTGAACGCT
	human GAPDH forward	GGATTTGGTCGTATTGGG
	human GAPDH reverse	GGAAGATGGTGATGGGATT
Plasmid construction	mouse HtrA1normal forward	ACGTCTCGAGGCCTCGCAGCGGTGACGAG
	mouse HtrA1 normal reverse	ACGTGAGCTCAGATGCAGCCCAATCTTCTCCTAACA
	mouse in/del forward	ACGTCTCGAGGCCTCGCAGCGGTGACGAG
	mouse in/del reverse	ACGTGAGCTCAGATGCAGCCCAATCTTCTCCTAACA
	human HTRA1-myc forward	ATGCACCGGTTGAGAGACACAAAAAATTCCAAC
	human HTRA1-myc reverse	ATGCGTCGACGTACAAGAAAGCTGGGCCCATGAGGCCT
	mouse Gtf2i-Flag forwmard (EcoRI)	ATGCGAATTCATGGCCCAAGTAGTGATGTCTGCCTTGCC
	mouse Gtf2i-flag reverse (XbaI)	ATGCTCTAGACCACGTGGGGTCTGGCTCCTGCTTGAT
Gft2i isofrom idendification	mouse Gtf2i isoform-F (TA cloning)	ATTCTGGCATTTCTCTGGAAATGGCCGC
	mouse Gtf2i isoform-F-A	AAAAGCTCCCCCTTTCGAAGGCTTTGCA
	mouse Gtf2i isoform-F-B	TCAGAAGGCAACGAGGGAACGGAAGTGG
	mouse Gtf2i isoform F-C	GTCCCTTCAGAAACCAGTGAGGACCCCG
	mouse Gtf2i isoform-R	TCCCAAGGCCTCGGCAAATTTCTTGCAG
Sequencing	Gtf2i-sequenceing-1	ATGGACTACAAAGACCATGACGGT
	Gtf2i-sequenceing-2	ATGGCCCAAGTAGTGATGTCTGCCT
	Gtf2i-sequenceing-3	GGTCCCTGTGCCATATGAGAAGATG
	Gtf2i-sequenceing-4	TGACTATTCTCCACCCACCAAGAGG
	Gtf2i-sequenceing-5	GATGGTTGACCAGCTCTTCTGCAAG
	Gtf2i-sequenceing-6	GAGAGGATTGTCCGTGGCAGCAATA
	Gtf2i-sequenceing-7	CTGAGATGTTCGAGACGGCCA
	Gtf2i-sequenceing-8	GTCCTTCAAAGCCCCCAGTTACCTG
	Gtf2i-sequenceing-9	CTACCACGTGGGGTCTGGCT
	Gtf2i-sequenceing-10	CAGGTAACTGGGGGCTTTGAAGGAC
	Gtf2i-sequenceing-11	TGGCCGTCTCGAACATCTCAG
	Gtf2i-sequenceing-12	TATTGCTGCCACGGACAATCCTCTC
	Gtf2i-sequenceing-13	CTTGCAGAAGAGCTGGTCAACCATC
	Gtf2i-sequenceing-14	CCTCTTGGTGGGTGGAGAATAGTCA
	Gtf2i-sequenceing-15	CATCTTCTCATATGGCACAGGGACC

Supplementary Table 5

Supplementary Table 5. EMSA probe sequence

No.	Name	Sequence	
		**Sense**	**Antisense**
1	Normal-1	GGTGTACCTGCTGTTAAAGGAGGTTACGACCTCTGATGCT	AGCATCAGAGGTCGTAACCTCCTTTAACAGCAGGTACACC
2	Normal-2	ATGCTGGGGTGGCCAGAGGGGATGGGAGTGGGTCTGGCAC	GTGCCAGACCCACTCCCATCCCCTCTGGCCACCCCAGCAT
3	Normal-3	GGCACTCTGAGGAAAGGGGGTGAAACCAGCTGAGAAGTCA	TGACTTCTCAGCTGGTTTCACCCCCTTTCCTCAGAGTGCC
4	Normal-4	GGCACTCTGAGGAAAGGGGGTGAAACCAGCTGAGAAGTCA	GAACCCTGGCTGGGGCCATGCCAGCAGGTAAAAGATGACT
5	Normal-5	GGCACTCTGAGGAAAGGGGGTGAAACCAGCTGAGAAGTCA	ACTCACCCATTCTCCCATAGCAACAGAACCCTGGCTGGGG
6	In/del-6	GGCACTCTGAGGAAAGGGGGTGAAACCAGCTGAGAAGTCA	ATAATTTAATTTTAGTTAATTAATTAATAACCGGGAGAGA
7	In/del-7	AATTATTTAGTTAATTTAATTAACTAAACTAATGGGTGAG	CTCACCCATTAGTTTAGTTAATTAAATTAACTAAATAATT

Uncropped raw western blot images

Fig. 1 c

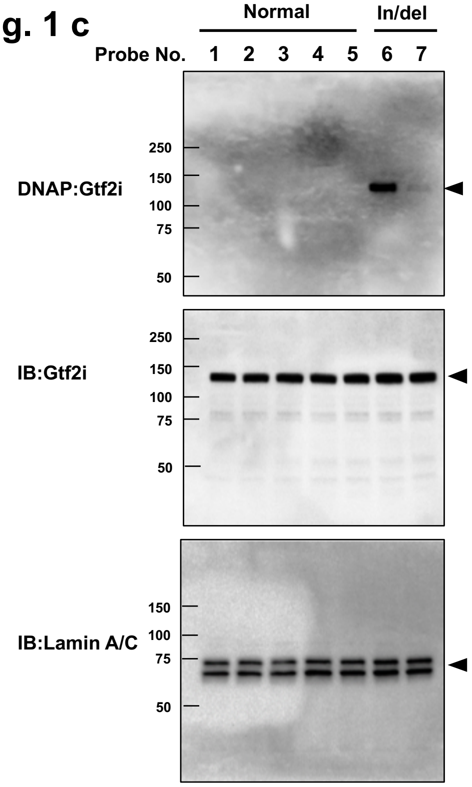

Fig. 1 g

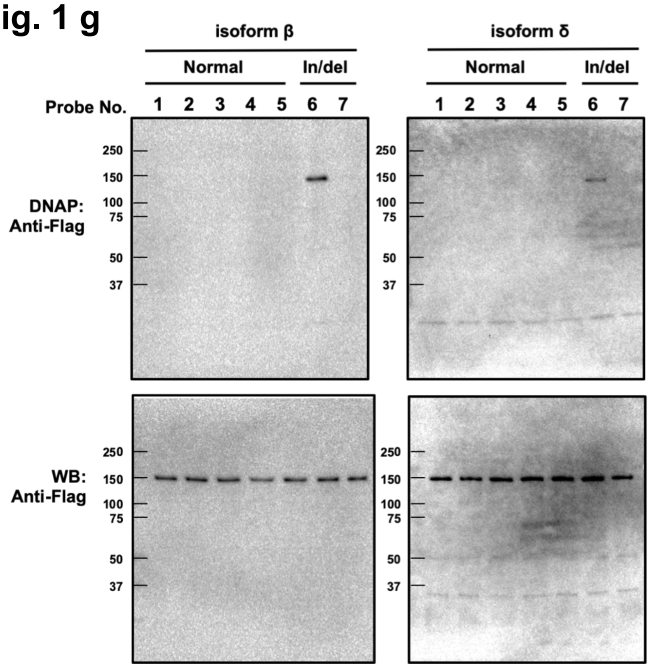

Fig. 1 h

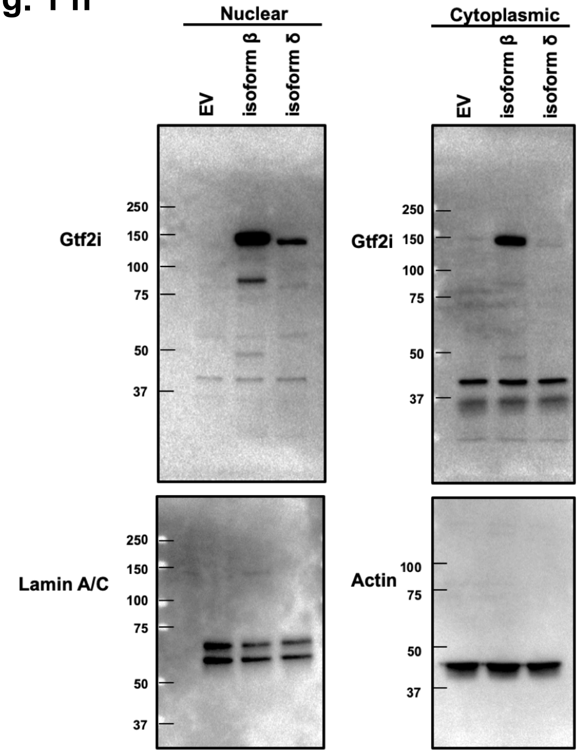

Fig. 2 c

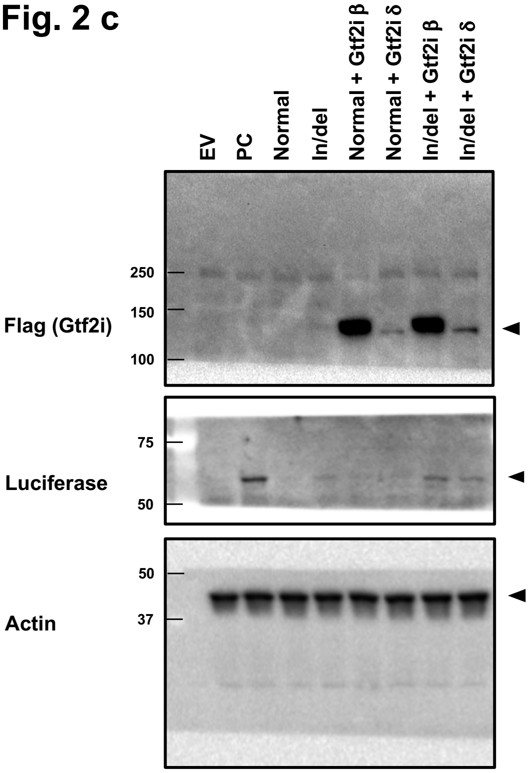

Uncropped raw western blot images

Fig. 5 a

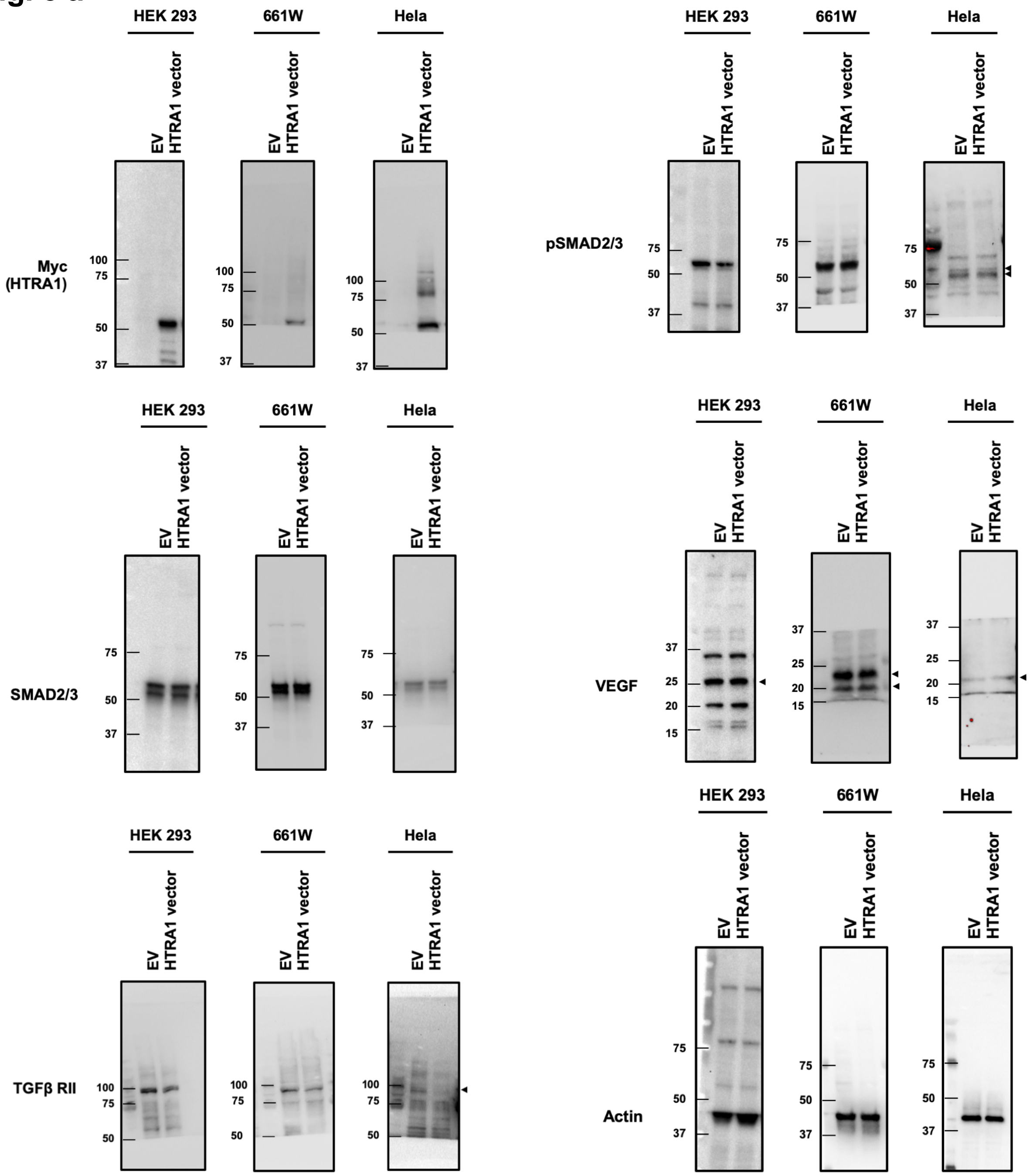

Fig. 5 c

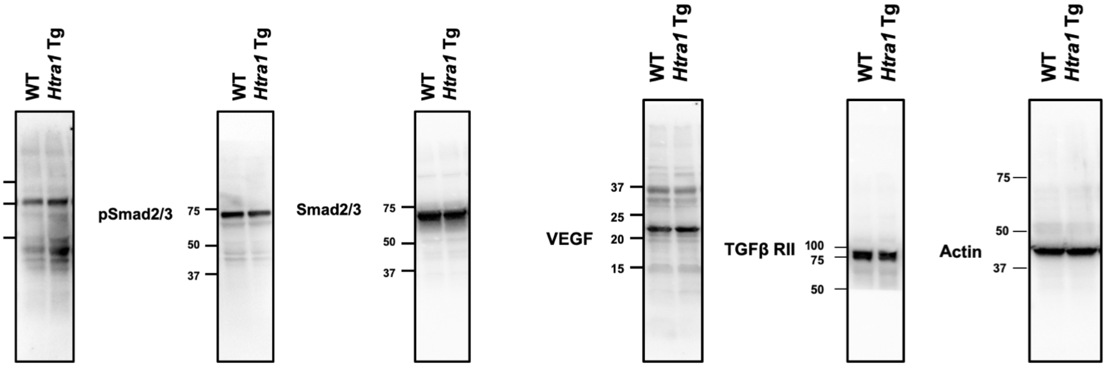

Supplement: Figures S1 to S9 and Tables S3 to S5 [file mmc1.pdf]
